# Patchouli alcohol triggers autophagic cell death in non-small cell lung cancer cells through targeting GNAI1 to dissociate the GNAI1/ARRB1 complex

**DOI:** 10.7150/ijbs.125690

**Published:** 2026-03-30

**Authors:** Sheng Zhang, Lei Tang, Yunzhou Pu, Yu You, Hongyu Chen, Yongqi Chen, Yanqing Huang, Xiaodie Liu, Qing Song, Qing Ji, Liu Yang

**Affiliations:** 1 Shanghai Baoshan District Wusong Central Hospital (Zhongshan Hospital Wusong Branch, Fudan University), Shanghai 200940, China.; 2 Guangdong Lung Cancer Institute, Guangdong Provincial People's Hospital (Guangdong Academy of Medical Sciences), Southern Medical University, Guangzhou 510080, China; Guangdong Provincial Key Laboratory of Translational Medicine in Lung Cancer, Guangdong Provincial People's Hospital (Guangdong Academy of Medical Sciences), Southern Medical University, Guangzhou 510080, China.; 3 Department of Medical Oncology & Cancer Institute of Integrative Medicine, Shuguang Hospital, Shanghai University of Traditional Chinese Medicine, Shanghai 201203, China.; 4 Chengdu University of Traditional Chinese Medicine, Chendu 610075, China.; 5 Department of Medical Oncology, Suzhou TCM Hospital Affiliated to Nanjing University of Chinese Medicine, Suzhou 215007, China.; 6 Department of Oncology, Baoshan Hospital Affiliated to Shanghai University of Traditional Chinese Medicine, Shanghai 201999, China.

**Keywords:** patchouli alcohol, autophagic cell death, non-small cell lung cancer, GNAI1, ARRB1

## Abstract

As the leading cause of global cancer-related mortality, lung cancer has a particularly poor prognosis among non-small cell lung cancer (NSCLC) subtypes, which constitute 85% of cases and are frequently diagnosed at advanced stages. Through integrated pharmacological approaches, this study elucidates the novel anti-NSCLC mechanism of patchouli alcohol (PA), a low-toxicity sesquiterpene alcohol. We confirmed that compared with conventional chemotherapeutics, PA induced autophagic cell death in NSCLC models (*in vitro*/*in vivo*) and resulted in superior biosafety profiles. Combining DARTS, molecular docking and CETSA, we subsequently identified GNAI1 as the direct molecular target of PA. ARRB1 was further characterized as the essential adaptor mediating PA/GNAI1-triggered autophagic cell death. Specifically, PA specifically disrupted the GNAI1-ARRB1 interaction, inhibiting downstream pro-survival pathways (ERK/JAK2-STAT3/mTOR) and ultimately leading to autophagic cell death in NSCLC cells. In summary, this work provides the first evidence that PA exerts anti-NSCLC effects by targeting the GNAI1/ARRB1 axis, offering a promising therapeutic strategy against advanced NSCLC.

## Introduction

Among all malignancies, lung cancer has the highest global incidence and mortality rates 1. Non-small cell lung carcinoma (NSCLC) constitutes 85-90% of all lung cancer cases 2. Current clinical interventions include surgery, chemotherapy, radiotherapy, targeted therapy, and immunotherapy. However, ‌locally advanced or metastatic disease is present in more than 70% of patients with NSCLC at the time of diagnosis 3, with a five-year relative survival rate of less than 24% despite multimodal treatments 4-5. Conventional chemotherapy has demonstrated limited tumor selectivity (20-30% efficacy) and pronounced toxicity, damaging both malignant and healthy tissues while inducing severe adverse effects that compromise patients' quality of life 6-7. Consequently, developing effective therapies for advanced NSCLC remains a critical research priority.

As a natural sesquiterpene alcohol, patchouli alcohol (PA) exhibits therapeutic potential for treating inflammatory bowel diseases, irritable bowel syndrome with diarrhea, and Alzheimer's disease 8-13. Nevertheless, its antitumor mechanisms remain incompletely characterized. Our preliminary studies revealed that PA dose-dependently inhibited proliferation across multiple cancer cell lines, with pronounced effects in NSCLC, although the underlying mechanisms remain unknown.

Macroautophagy (hereafter termed autophagy) is an evolutionarily conserved cellular self-digestion mechanism that is ubiquitous in eukaryotic systems 14. This process begins with the formation of double-membraned autophagosomes that sequester cytoplasmic components, which subsequently fuse with lysosomes to form autolysosomes, culminating in cargo degradation and component recycling 15. As a key marker of autophagy, Beclin-1 governs autophagosome initiation and assembly, while microtubule associated protein light chain 3 (LC3) mediates autophagosomal membrane elongation, and p62 facilitates the degradation of selective autophagic cargo 16. In oncological contexts, autophagy has dual functions: it sustains tumor growth by providing energy and nutrients during stress but paradoxically suppresses malignancy through the clearance of damaged organelles and carcinogenic aggregates 17. Pharmacologically induced excessive autophagy transcends homeostatic recycling, triggering programmed autophagic cell death (ACD), which is characterized by unrestrained autophagic flux 18. This ACD modality is often concurrent with apoptosis and represents a potent tumor-suppressive pathway wherein autophagy transitions from a “cellular cleaner” to a tumor-eliminating mechanism, offering novel therapeutic targets for oncology 19. GNAI1 (Gαi), a member of the G protein family, serves as a signal transducer that relays messages from activated G protein-coupled receptors (GPCRs) to intracellular effectors. This process modulates signaling cascades that regulate cell death and differentiation 20-21. Additionally, GNAI1 interacts with other signaling molecules, including ARRB1 (β-arrestin1), which is involved in receptor desensitization and internalization 22. These interactions further influence multiple signaling pathways, underscoring the pivotal role of GNAI1 in maintaining cellular responsiveness to external stimuli 23. Moreover, these findings offer valuable insights into potential novel therapeutic strategies for cancer management.

Therefore, building on the established growth-inhibitory effects of PA on NSCLC cells, this study aims to decipher its contemporary pharmacological mechanisms through the lens of autophagic cell death. Specifically, we will identify molecular targets‌ of PA that mediate autophagic cell death pathways, delineate key signaling axes‌ orchestrating autophagic execution, and establish a translational foundation‌ for future clinical deployment of PA-based therapies.

## Results

### PA induces autophagic cell death in NSCLC cells

**Figure 1:**
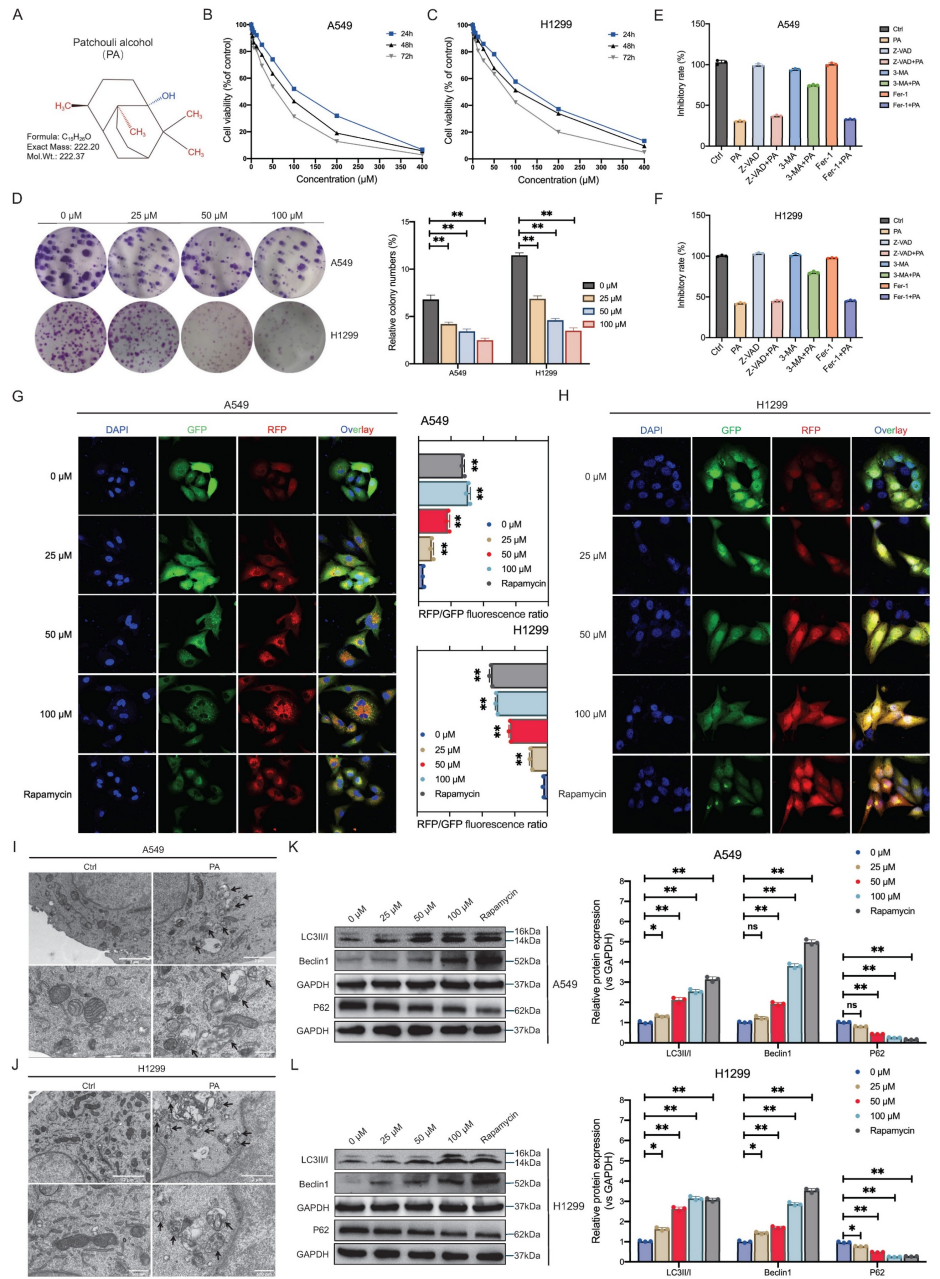
PA induces autophagic cell death in NSCLC cells. A. The two-dimensional chemical structure of PA. B-C. CCK-8 method was used to detect the inhibitory effect of PA on the proliferation of NSCLC cell lines A549 and H1299. D. Plate clone formation assay was used to detect the inhibitory effect of PA on NSCLC cell clone formation. E-F. CCK-8 assays was used to detect the inhibitory effect of PA on the proliferation of NSCLC cell lines A549 and H1299 in the presence of autophagy inhibitor 3-MA, apoptosis inhibitor Z-VAD, and ferroptosis inhibitor Fer-1. G-H. The RFP-GFP-LC3 dual fluorescence reporting system was used to detect the effect of PA on autophagy in NSCLC cell lines A549 and H1299, with a scale of 10 μm. I-J. Transmission electron microscopy was used to detect the effect of PA on autophagy in NSCLC cell lines A549 and H1299, with a scale of 2 μm in the upper figure and 500 nm in the lower figure. K-L. Western blot was used to detect the effect of PA on the expression of autophagy related proteins Beclin-1 and LC3A/B in NSCLC cell lines A549 and H1299. Compared with the control group, * *P* < 0.05, **P < 0.01.

‌PA, a natural product with a defined sesquiterpene structure (Fig. 1A), exhibits anti-NSCLC activity, although its precise pharmacological mechanism remains incompletely understood. A time-course analysis identified 48 hours as the optimal intervention window for PA. Dose-response curves revealed half-maximal inhibitory concentrations (IC50s) of 92.49 μM for A549 cells and 107.9 μM for H1299 cells (Fig. 1B-C). Consequently, in subsequent *in vitro* experiments, the dose-dependent effects of PA on NSCLC cells at low (25 μM), medium (50 μM), and high (100 μM) concentrations were systematically investigated. Subsequent plate colony formation assays further confirmed that PA dose-dependently inhibited NSCLC cell proliferation (Fig. 1D).

To elucidate the primary mechanism by which PA induces NSCLC cell death, we conducted CCK-8 assays in the presence of the autophagy inhibitor 3-MA, the apoptosis inhibitor Z-VAD, and the ferroptosis inhibitor Fer-1 to elucidate the main pathway of PA activity. The results revealed that PA primarily inhibited NSCLC cell proliferation by inducing autophagic death and had a low-degree pro-apoptotic effect on NSCLC cells at the current PA dose, but its role in promoting ferroptosis was not determined (Fig. 1E-F). Although flow cytometry demonstrated that PA partially induced apoptosis in NSCLC cells (Fig. S1A, S1B), the effect of PA on CRC cell apoptosis was far less significant than its effect on autophagic cell death in CRC cells. The RFP-GFP-LC3 dual-fluorescence reporter system subsequently demonstrated a dose-dependent increase in autolysosomes, indicating robust autophagic flux induction and autophagic cell death in NSCLC cells (Fig. 1G-H). Moreover, compared with those of the controls, the TEM pictures revealed significant increases in the numbers of autophagosomes and autolysosomes in the PA-treated cells, indicating enhanced autophagic activity (Fig. 1I-J).

Current evidence indicates that Beclin-1 upregulation reflects autophagy initiation, whereas increased LC3-II expression is positively correlated with autophagosomal membrane expansion. In addition, the degradation of P62, a selective autophagy adaptor protein, indicates the complete activation of autophagic flux. The membrane localization and accumulation of LC3-II, coupled with the upregulation of Beclin-1 expression, jointly drive autophagosome formation, whereas the simultaneous degradation of p62 confirms the effective clearance of autophagic substrates via lysosomes. Dynamic changes in these three factors constitute the typical characteristics of complete autophagic flux activation. Our western blot results demonstrated that PA upregulated the expression of Beclin-1 and LC3-II in a dose-dependent manner while simultaneously downregulating the expression of P62 (Fig. 1K-L). These synergistic changes in the expression of the above three markers support the notion that PA-mediated activation of canonical autophagy signaling via autophagosome biogenesis ultimately induces autophagic cell death in NSCLC cells.

### PA inhibits NSCLC tumor growth by inducing autophagic cell death

**Figure 2:**
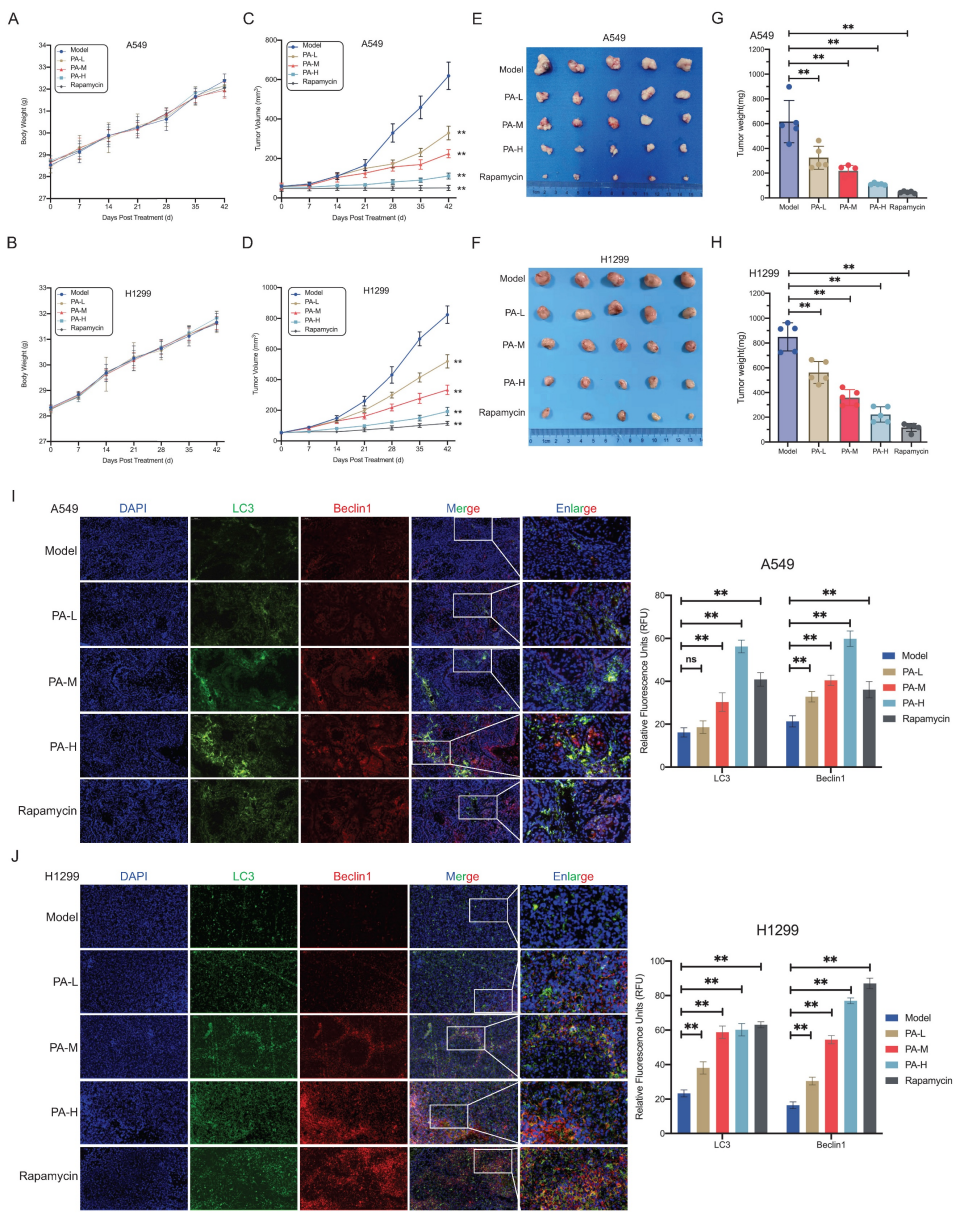
PA inhibits NSCLC tumor growth by inducing autophagic cell death. A-B. The effect of PA on the body weight of subcutaneous transplanted tumor nude mice in each group. A: A549, B: H1299. C-D. The effect of PA on the volume of subcutaneous transplanted tumors in nude mice in each group. C: A549, D: H1299. E-F. Subcutaneous transplanted tumors in nude mice in each group. E: A549; F: H1299. G-H. The effect of PA on the final weight of subcutaneous transplanted tumors in each group of nude mice. G: A549, H: H1299. I-J. Tissue immunofluorescence detection of the effect of PA on autophagy related protein expression in tumor tissues of nude mice in each group. Scale: 200 μm. Compared with the model group, * *P* < 0.05, * * *P* < 0.01.

To evaluate the *in vivo* antitumor efficacy of PA, we established subcutaneous NSCLC xenograft models in nude mice. Body weight trajectories gradually increased across all groups, with no statistically significant intergroup differences (Fig. 2A-B). Tumor volume monitoring revealed that low- (25 mg/kg), medium- (50 mg/kg), and high-dose (100 mg/kg) PA treatment significantly inhibited tumor growth compared with that in the model group, with dose-dependent suppression of tumor expansion (Fig. 2C-D). After 42 days of intervention, the excised tumor masses demonstrated dose-dependent reduction (Fig. 2E-H). These data confirm the *in vivo* dose-responsive antitumor activity of PA in NSCLC xenografts. No treatment-related mortality or adverse effects (e.g., diarrhea, lethargy, or reduced feeding) were observed. Liver function assessments further supported the *in vivo* safety profile of PA at therapeutic concentrations (Fig. S2A, S2B).

Given *in vitro* evidence of PA-induced autophagic cell death, we analyzed key autophagy markers in tumor tissues. In A549-derived xenografts, the LC3 fluorescence intensity increased in the medium- and high-dose PA groups, and Beclin-1 expression was upregulated across all the PA-treated groups (Fig. 2I). H1299-derived xenografts exhibited dose-dependent upregulation of both LC3 and Beclin-1 expression (Fig. 2J). These biomarker modulations mirrored the patterns observed with the autophagy inducer rapamycin (Fig. 2I-J). Therefore, combined *in vitro* and *in vivo* evidence demonstrates that PA activates functional autophagic flux through core autophagy regulators (Beclin-1/LC3), triggering autophagic cell death and consequent NSCLC tumor suppression.

### PA targets GNAI1 to induce autophagic cell death in NSCLC cells

PA is a sesquiterpene compound with the chemical formula C₁₅H₂₆O and a molecular weight of 222.37 g/mol. Its two-dimensional structure (Fig. 1A, generated using KingDraw software) reveals a characteristic tricyclic skeleton and a hydroxyl group. The tight binding observed between PA and its target protein likely arises from the combined effects of several factors: highly complementary molecular shapes, precisely matched charge distributions, and potential hydrogen- bond formation. This specific mode of molecular interaction likely underpins the broad spectrum of biological activities of PA.

**Figure 3:**
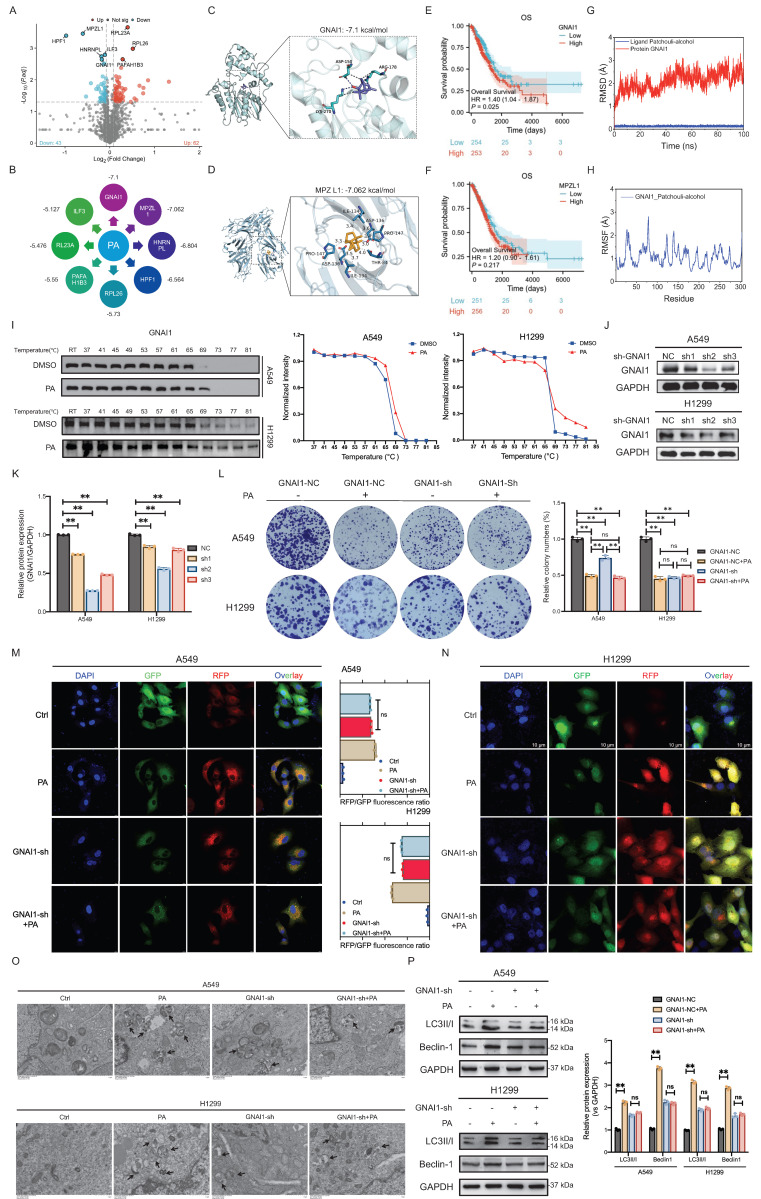
PA targets GNAI1 to induce autophagic cell death in NSCLC cells. A. DARTS analysis identifies potential drug targets for PA, and differential proteins are represented in the form of volcano plots. B. The top 8 potential drug target proteins for PA are MPZL1, RL23A, HPF1, RPL26, HNRNPL, ILF3, GNAI1, and PAFAH1B3, each of which is labeled with binding free energy. C. Molecular docking results of PA and GNAI1. D. Molecular docking results of PA and MPZL1. E. Analyzing the relationship between GNAI1 expression and OS of lung adenocarcinoma patients based on the TCGA database. F. Analyzing the relationship between MPZL1 expression and OS of lung adenocarcinoma patients based on the TCGA database. G-H. Molecular dynamics simulation results of PA and GNAI1. I. CETSA experiment verifies the binding effect between PA and GNAI1. J-K. WB detection and quantitative analysis of gene knockdown efficiency of GNAI1 in A549 and H1299 cells. L. Observe the effect of PA on the colony forming ability of NSCLC cells after knocking down GNAI1. M-N. The RFP-GFP-LC3 dual fluorescence reporting system was used to detect the effect of PA on autophagy in NSCLC cell lines A549 and H1299 after knocking down GNAI1, with a scale of 10 μm. O. Transmission electron microscopy was used to detect the effect of PA on autophagy in NSCLC cell lines A549 and H1299 after knocking down GNAI1, with a scale of 1 μm. P. Observation of the effect of PA on autophagy related protein expression in NSCLC cells after knocking down GNAI1. Compared with the negative control group, *P < 0.05, **P < 0.01.

To identify potential target proteins of PA, we employed DARTS technology in the NSCLC cell line A549. Based on the results of the volcano plot analysis, we identified 105 differentially expressed proteins between the groups, with 62 upregulated and 42 downregulated (Fig. 3A). The top 8 differentially expressed proteins included MPZL1, RL23A, HPF1, RPL26, HNRNPL, ILF3, GNAI1, and PAFAH1B3 (Fig. 3B). Batch screening of differential proteins was conducted using molecular docking to evaluate interactions based on binding free energy, hydrophobic interactions, and hydrogen bonding networks. The results revealed that GNAI1 (-7.1 kcal/mol) and MPZL1 (-7.062 kcal/mol) exhibited the highest binding affinity with PA: the key binding sites for GNAI1 were ASP-150 (hydrogen bond/hydrophobic), ARG-178 (hydrophobic), and LYS-270 (hydrogen bond/hydrophobic), while MPZL1 formed a stable complex via PRO-147 (hydrophobic) and ASP-136 (hydrogen bond/hydrophobic) (Fig. 3C-D). Among the other candidates, HNRNPL (-6.804 kcal/mol) and HPF1 (-6.564 kcal/mol) met the screening threshold (ΔG ≤ -6.5 kcal/mol), whereas ILF3 (-5.127 kcal/mol) showed the weakest binding (Fig. S3A-S3F, Fig. 3B). Therefore, based on the free energy ranking and key interaction site analysis, GNAI1, MPZL1, HNRNPL, and HPF1 were identified as candidate targets for further validation.

An analysis of survival data for lung adenocarcinoma (LUAD) from the TCGA database revealed that patients with high GNAI1 expression had significantly worse overall survival (OS) than those with low- GNAI1 expression did (HR = 1.4, *P* < 0.05). In contrast, the MPZL1 expression level was not significantly correlated with OS (HR = 1.3, *P* > 0.05) (Fig. 3E-F). These findings identify high GNAI1 expression as a predictor of poor prognosis in patients with NSCLC and support its candidacy as a therapeutic target. In pharmacokinetic assessments, the root mean square deviation (RMSD) is used to quantify the conformational dynamics of protein-ligand complexes during simulations. Elevated RMSD values indicate increased conformational flexibility, whereas lower fluctuations reflect structural stability. Molecular dynamics simulations (100 ns) demonstrated stable average RMSD values (< 3.0 Å) for all the PA-candidate target complexes (GNAI1, MPZL1, HNRNPL, and HPF1). Among these, MPZL1 and GNAI1 exhibited minimal residue fluctuation (RMSD = 1-2 Å) at binding sites, although GNAI1 displayed localized conformational adaptation at key residues (e.g., ARG-178) (Fig. 3G, Fig. S4A-S4C). Consistent with the results of the free energy calculations, the binding affinity of the MPZL1-PA complex was the strongest, followed by that of GNAI1-PA, whereas those of the HNRNPL-PA and HPF1-PA complexes were weaker (Fig. 3G, Fig. S4D-S4F). Integrating these findings, we identified MPZL1 and GNAI1 as the most promising binding targets for PA.

To further validate the ideal target of PA, we employed CETSA to assess binding. The results demonstrated significant thermal stabilization of the GNAI1 protein in the PA-treated groups compared with those in the controls across the A549 and H1299 cell lines following thermal challenge (Fig. 3H), confirming that the formation of the PA-GNAI1 complex enhanced protein stability. In contrast, PA failed to stabilize the MPZL1 or HNRNPL proteins (Fig. S5A-S5B), further supporting the target specificity of GNAI1. Having established GNAI1 as a direct binding target, we investigated whether PA modulates GNAI1 expression. Quantitative analysis revealed no significant alterations in GNAI1 mRNA or protein levels following PA treatment (Fig. S6A-S6D), indicating that the bioactivity of PA occurs through post-translational mechanisms rather than transcriptional/translational regulation.

To investigate whether PA modulates autophagic cell death in NSCLC cells through GNAI1, we established a stable GNAI1-knockdown NSCLC cell line using lentiviral infection technology (Fig. 3I-J). We then assessed the effects of PA on cell growth and autophagic death after GNAI1-knockdown. The results demonstrated that PA significantly inhibited colony formation in GNAI1-competent cells. Upon GNAI1 knockdown, basal colony formation substantially decreased, confirming the essential role of GNAI1 in sustaining tumor proliferation. Crucially, following GNAI1 knockdown, compared with no treatment, PA treatment no longer significantly suppressed colony formation (Fig. 3K-L), indicating that the antitumor efficacy of PA requires functional GNAI1. These findings establish GNAI1 as both a critical NSCLC proliferation regulator and a core pharmacological target of PA.

To determine whether GNAI1 mediates PA-induced autophagic cell death in NSCLC, we examined the effects of PA on autophagy following GNAI1 knockdown. RFP-GFP-LC3 dual-fluorescence reporter system (Fig. 3M-N) and TEM analysis (Fig. 3O) demonstrated that compared with no treatment, GNAI1 knockdown alone substantially induced autophagy in NSCLC cells; however, after GNAI1 was knocked down, PA treatment failed to significantly increase autophagy (Fig. 3M-O). Moreover, in control NSCLC cells, PA significantly decreased Beclin-1 protein levels while inducing LC3-II accumulation (Fig. 3P), indicating that autophagic flux was activated. GNAI1 knockdown alone substantially reduced basal Beclin-1 and LC3-II expression, further confirming the essential role of GNAI1 in the regulation of autophagy in NSCLC. Crucially, after GNAI1 knockdown, compared with no treatment, PA treatment failed to significantly modulate either Beclin-1 or LC3-II levels (Fig. 3P). These data demonstrated that PA-induced autophagic death requires functional GNAI1.

### PA dissociates the GNAI1/ARRB1 complex to induce autophagic cell death in NSCLC

Collectively, these results demonstrate that GNAI1 serves as a critical target for PA despite its unaffected expression levels, suggesting that PA modulates GNAI1 function through noncanonical mechanisms. To identify potential mediators, we performed protein-protein interaction analysis using the STRING database (v11.5). This revealed high-confidence interactors of GNAI1 including ARRB1, SCARB2, CALM3, FTL, and RAB11A, with ARRB1 showing the strongest binding association (Fig. S7). Critically, published experimental evidence confirms the direct GNAI1-ARRB1 interaction, establishing ARRB1 as our primary candidate for further investigation.

**Figure 4:**
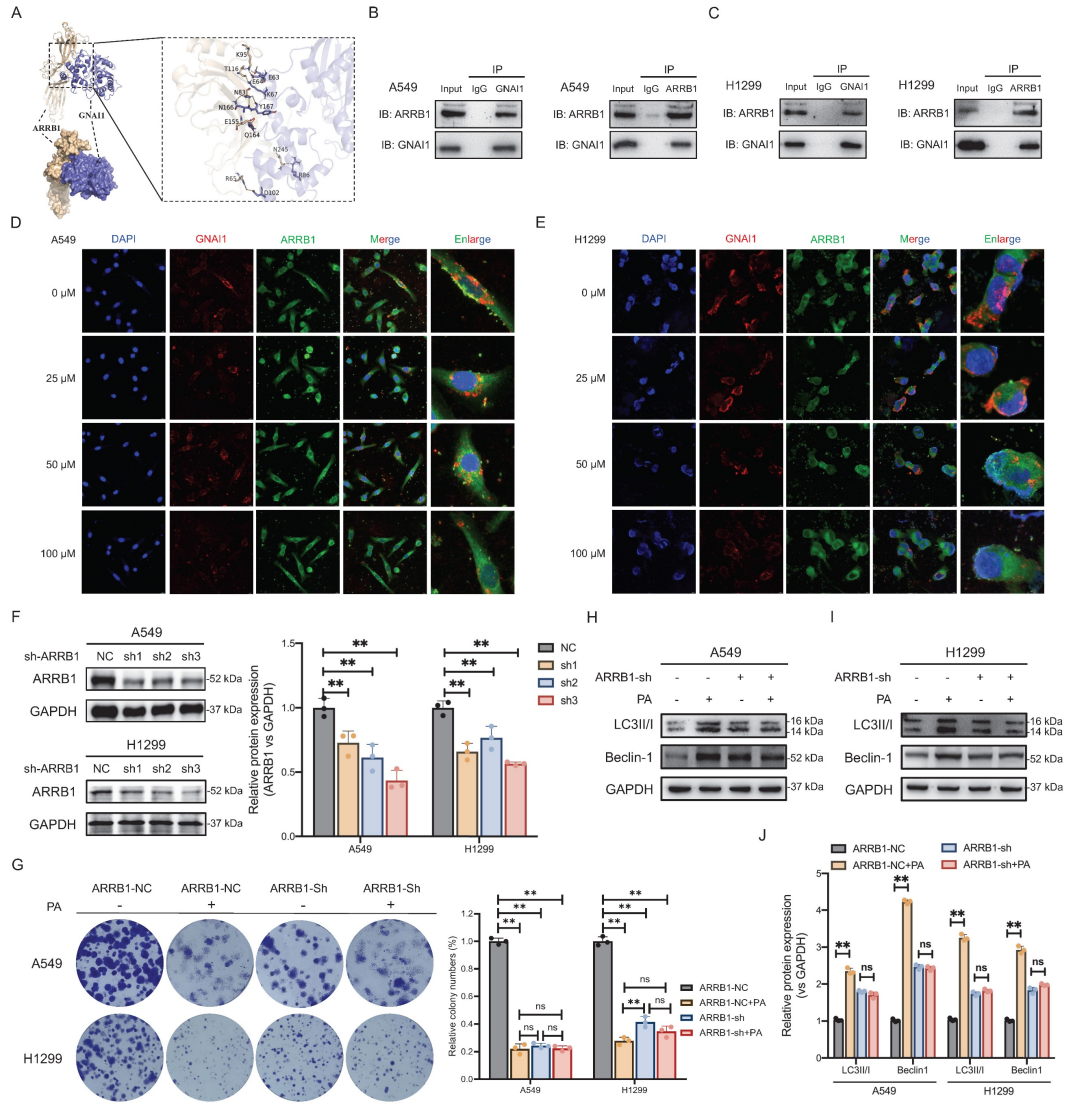
PA dissociates GNAI1/ARRB1 complex to induce autophagic cell death in NSCLC. A. ZDOCK 3.0.2 predicts the binding mode of ARRB1-GNAI1 complex. The wheat color is ARRB1, blue is GNAI1 protein, and the yellow dashed line represents hydrogen bonding. B-C. Co-IP experiment verifies the protein interaction between GNAI1 and ARRB1 in NSCLC A549 and H1299 cell lines. D-E. Immunofluorescence co-localization analysis of GNAI1 and ARRB1 protein interaction after PA intervention at different concentrations in A549 and H1299 cells, with a scale of 20 μm. F. Western blot detection of gene knockout efficiency of ARRB1 in A549 and H1299 cells. G. Knockdown ARRB1 and observe the effect of PA on the colony forming ability of NSCLC cells. H-I-J. Knockdown ARRB1 and observe the effect of PA on autophagy related protein expression in NSCLC cells. Compared with the negative control group, *P<0.05, **P<0.01.

To determine whether GNAI1 directly interacts with ARRB1, we performed molecular docking simulations using ZDOCK 3.0.2 to predict binding affinity and interaction modes. The results of the computational analysis revealed strong binding potential between GNAI1 and ARRB1, with a calculated binding energy of -16.0 kcal/mol. Furthermore, ZDOCK predicted specific hydrogen bonding interactions stabilizing the complex: residues K95, T116, N83, E155, R65, and N245 on ARRB1 formed bonds with E63, E64, K67, Y167, N166, Q164, R86, and D102 on GNAI1 (Fig. 4A).

To validate the endogenous GNAI1-ARRB1 interaction, we performed Co-IP in NSCLC cell lines. This demonstrated direct binding between natively expressed proteins under physiological conditions (Fig. 4B-C). Subsequent immunofluorescence imaging confirmed significant the subcellular colocalization of GNAI1 and ARRB1 (Fig. 4D-E), indicating that spatial proximity is consistent with functional interaction. To assess the functional impact of PA, we treated cells with increasing concentrations of PA. Importantly, PA dose-dependently reduced GNAI1-ARRB1 colocalization (Fig. 4D-E), suggesting that compound-mediated disruption of this interaction occurred. These findings position ARRB1 as a critical adaptor in PA-induced autophagic cell death and indicate that PA likely disrupts autophagic signaling by modulating the GNAI1-ARRB1 interface.

To elucidate the role of ARRB1 in PA-mediated autophagic cell death in NSCLC, we first confirmed that PA does not directly modulate ARRB1 expression (mRNA/protein: Fig. S8A-S8F) or bind to the ARRB1 protein (Fig. S9A-S9B). Lentiviral-mediated ARRB1 knockdown (Fig. 4F) subsequently revealed two critical functions. First, colony formation significantly decreased in ARRB1-knockdown NSCLC cells, indicating that ARRB1 maintains tumor proliferation. Second, PA suppressed colony formation in control cells but had negligible effects on ARRB1-knockdown cells, demonstrating that ARRB1 mediates the antitumor effect of PA (Fig. 4G). These results establish ARRB1 as both an essential proliferative regulator and an indispensable mediator of PA-induced autophagic cell death via GNAI1 targeting.

To determine the role of ARRB1 as a critical mediator of PA-induced autophagic cell death in NSCLC, we analyzed the expression of autophagy markers (Beclin-1 and LC3-II) following ARRB1 knockdown. The results demonstrated that, in ARRB1-intact cells, PA treatment significantly reduced Beclin-1 expression and increased LC3-II accumulation, confirming autophagic flux activation (Fig. 4H-J). ARRB1 knockdown alone decreased basal Beclin-1 and LC3-II expression, establishing its essential role in autophagic cell death regulation. Crucially, in NSCLC cells with ARRB1 knockdown, compared with no treatment, PA treatment resulted in no statistical difference in the expression of Beclin-1 or LC3-II (Fig. 4H-J), confirming that ARRB1 is indispensable for the ability of PA to induced autophagic cell death in NSCLC cells.

### ERK and mTOR signaling pathways represent primary downstream effectors of PA-induced autophagic cell death via targeting‌ GNAI1/ARRB1

Building on our demonstration that PA triggers autophagic cell death in NSCLC by disrupting the GNAI1/ARRB1 complex, we next sought to identify the specific downstream signaling pathways that mediate this effect. Given the established involvement of multiple signaling cascades in autophagy regulation 24-26, we systematically investigated the molecular networks associated with GNAI1 and ARRB1 to elucidate the underlying mechanism of PA.

**Figure 5:**
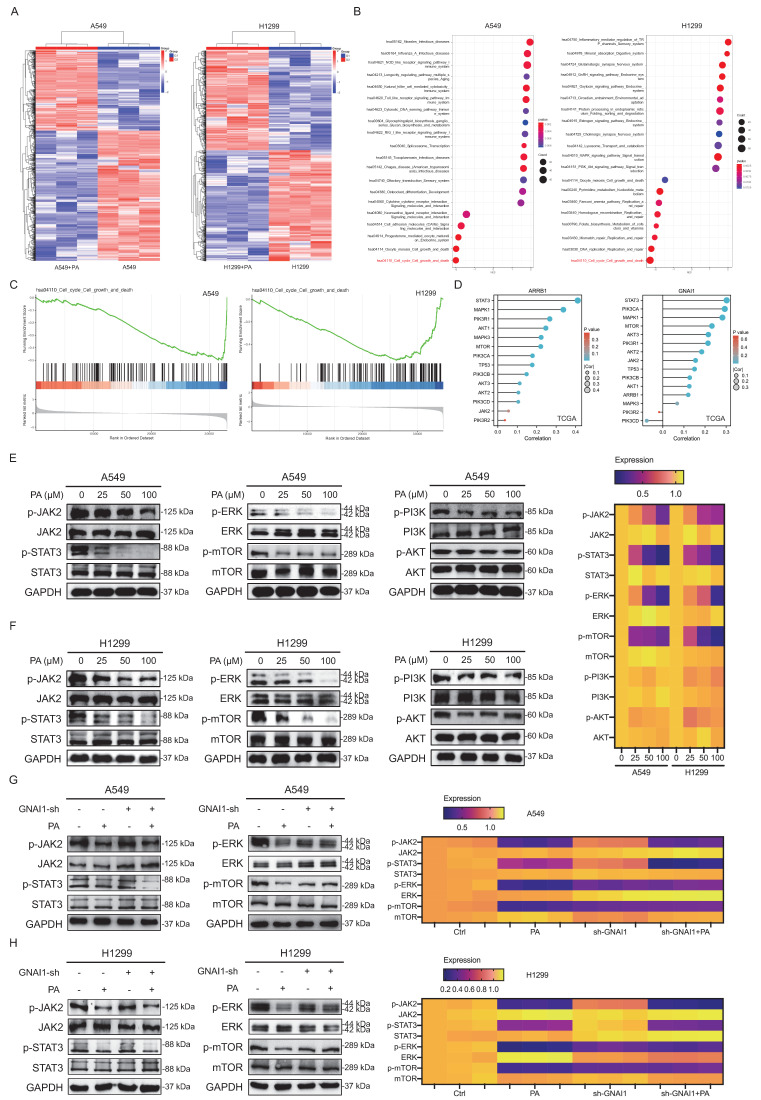
ERK and mTOR signaling pathways represent primary downstream effectors of PA-induced autophagic cell death via targeting GNAI1/ARRB1‌. A. Transcriptomic analysis of the effect of PA on mRNA expression profiles in NSCLC cell lines A549 and H1299, with differential expression represented in the form of heat maps. B. GSEA enrichment analysis of transcriptome differentially expressed genes after PA intervention (left: A549, right: H1299), presented in the form of a bubble plot. C. GSEA enrichment analysis of transcriptome differentially expressed genes related to cell cycle, growth, and death pathways (left: A549, right: H1299). D. Mining cell growth and death signaling pathways closely associated with ARRB1 and GNAI1 based on the TCGA database (left: A549, right: H1299). E-F. Western blot detection of the effects of different concentrations of PA intervention on cell growth and death related signaling pathways in NSCLC cell lines A549 and H1299. Quantitative results are presented in the form of heat maps. G-H. Western blot detection of the effect of PA on cell growth and death related signaling pathways in NSCLC cell lines A549 and H1299 after knocking down GNAI1. Compared with the negative control group, *P<0.05, **P<0.01.

Transcriptomic profiling of PA-treated versus control NSCLC cells (Fig. 5A) revealed differentially expressed genes whose expression was strongly enriched in cell cycle regulation, proliferation, and cell death pathways across both the A549 and H1299 lines. KEGG/GO analyses revealed the significant involvement of multiple signaling axes, including the JAK2/STAT3, AMPK/mTOR, PI3K/AKT, MAPK/ERK1/2, and p53/mTOR pathways (Fig. 5B-C). These findings suggest that PA inhibits NSCLC proliferation through synergistically inhibiting pro-proliferative signaling pathways and promoting autophagy-related gene expression. Leveraging the TCGA-LUAD data, we subsequently quantified the correlations between GNAI1/ARRB1 expression and these identified pathways. Significant associations emerged with the JAK2/STAT3, MAPK/ERK1/2, and PI3K/AKT/mTOR signaling axes (Fig. 5D), indicating that GNAI1/ARRB1-mediated autophagy operates through these core effector pathways.

To validate these predictions, we systematically assessed key signaling proteins including the JAK2/STAT3, MAPK/ERK1/2, PI3K/AKT, and mTOR pathways, to identify the specific effectors that mediate PA-induced autophagic cell death via GNAI1/ARRB1 targeting. Western blot analysis revealed dose-dependent reductions in phosphorylated JAK2, STAT3, ERK, and mTOR in both the A549 and H1299 cell lines following PA treatment, whereas PI3K and AKT phosphorylation remained unaffected (Fig. 5E-F). These results establish ERK, JAK2/STAT3, and mTOR as primary downstream mediators of PA-driven autophagic death in NSCLC cells through GNAI1/ARRB1 regulation.

To investigate whether PA exerts its inhibitory effects on the aforementioned signaling pathways directly or indirectly through targeting GNAI1/ARRB1, we examined the impact of PA on these pathways following GNAI1 knockdown. The results demonstrated that after GNAI1 knockdown, the activation of ERK and mTOR signaling was significantly suppressed. Moreover, the inhibitory effect of PA on ERK and mTOR signaling was markedly attenuated following GNAI1 knockdown (Fig. 5G-H). However, the activity of the JAK2 and STAT3 signaling pathways remained unaffected by GNAI1 knockdown, and PA robustly inhibited these pathways even after GNAI1 knockdown (Fig. 5G-H). These findings suggest that PA can simultaneously inhibit the JAK2/STAT3, ERK, and mTOR signaling pathways as a whole. However, it appears that PA primarily inhibits the ERK and mTOR signaling pathways by targeting GNAI1. The precise mechanism underlying the inhibition of the JAK2/STAT3 signaling pathway by PA warrants further in-depth exploration and clarification.

### GNAI1 is required for PA-mediated suppression of NSCLC xenograft growth‌

Having established GNAI1 as the primary molecular target of PA-induced autophagic cell death *in vitro*, we next sought to validate its functional significance *in vivo*. By employing subcutaneous xenograft models generated from either wild-type NSCLC cells or GNAI1-knockdown NSCLC cells, we assessed the antitumor efficacy of PA across both models.

**Figure 6:**
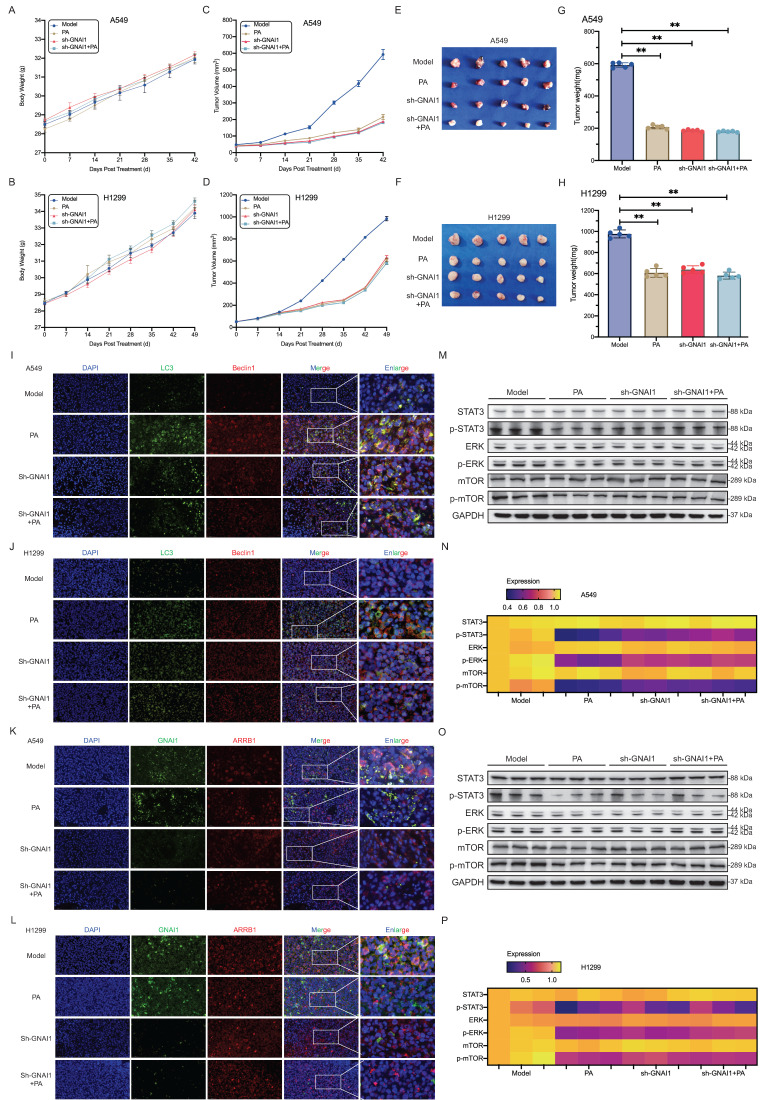
GNAI1 is required for PA-mediated suppression of NSCLC xenograft growth‌. A-B. Knockdown GNAI1 and observe the effect of PA on the body weight of subcutaneous transplanted tumor nude mice in each group. A: A549, B: H1299. C-D. After knocking down GNAI1, observe the effect of PA on the volume of subcutaneous transplanted tumors in nude mice in each group. C: A549, D: H1299. E-F. Subcutaneous transplanted tumors in nude mice in each group. E: A549, F: H1299. G-H. Observation of the effect of PA on the final weight of subcutaneous transplanted tumors in nude mice after knockdown of GNAI1. G: A549, H: H1299. I-J. After knocking down GNAI1, tissue immunofluorescence was used to detect the effect of PA on autophagy related protein expression in tumor tissues of nude mice in each group. Scale: 50 μm. K-L. After knocking down GNAI1, tissue immunofluorescence was used to detect the protein interaction between GNAI1 and ARRB1 in tumor tissues of nude mice in each group. Scale: 50 μm. M-N-O-P. After knocking down GNAI1, Western blot was used to detect the effect of PA on autophagy related signaling pathway proteins in tumor tissues of nude mice in each group (A549 or H1299). Compared with the model group, *P < 0.05, **P < 0.01.

Consistent with standard preclinical protocols, we first monitored longitudinal changes in mouse body weight. All groups showed progressive weight gains during the study, and there were no statistically significant differences between treatment cohorts (Fig. 6A and 6E), indicating the absence of systemic toxicity. Measurements of tumor volume revealed that compared with no knockdown, PA treatment significantly inhibited xenograft growth (Model group). Notably, GNAI1 knockdown alone substantially reduced tumor growth kinetics, with efficacy modestly exceeding that of PA monotherapy. Crucially, compared with GNAI1 knockdown alone, the combination of PA with GNAI1 knockdown did not result in any additional antitumor benefit (Fig. 6B and 6F). Upon study termination and tumor excision, the terminal tumor weights corroborated the following findings. Compared with the model group, both the PA-treated and GNAI1-knockdown groups had significantly reduced tumor masses. However, tumors from the combination treatment cohort exhibited no further reduction in size or weight relative to those from the GNAI1-knockdown cohort (Fig. 6C, 6D, 6G, 6H). These findings establish GNAI1 as the essential mediator of PA-induced autophagic cell death in NSCLC tumors.

Building upon these findings, we further investigated the central role of GNAI1 in PA-induced autophagic cell death using harvested xenograft tissues. Immunofluorescence analysis revealed that compared with the model group, GNAI1 knockdown moderately enhanced autophagic flux, but the combination treatment (PA + GNAI1 knockdown) failed to increase autophagic death (Fig. 6I-J). Crucially, GNAI1/ARRB1 complex formation was significantly reduced in GNAI1 knockdown tumors compared with the model group. However, compared with GNAI1 knockdown alone, PA co-administration produced no additional disruption of the complex formation (Fig. 6K-L). These collective observations prompted us to examine how PA modulates downstream signaling pathways and autophagic activation specifically under GNAI1-deficient conditions. Western blot analysis revealed that PA treatment ‌reduced‌ the phosphorylation of STAT3, ERK, and mTOR in NSCLC subcutaneous xenografts. However, compared with GNAI1 knockdown alone, ‌combination treatment with PA and GNAI1 knockdown did not further reduce‌ the phosphorylation levels of STAT3, ERK, and mTOR (Fig. 6M-N). ‌Collectively, these results indicate that GNAI1 is essential for the PA-mediated suppression of NSCLC xenograft growth.

## Discussion

Globally, lung cancer continues to be the primary cause of cancer-related deaths, with NSCLC accounting for approximately 85% of all cases 1. The development of novel therapeutic agents for patients with advanced NSCLC represents a critical unmet medical need, particularly for those who develop resistance to conventional therapies.

As a naturally occurring sesquiterpene compound derived from Pogostemon cablin, PA has demonstrated multifaceted pharmacological activities across various disease models 8-13. Emerging evidence suggests its therapeutic potential in inflammatory bowel disease (IBD) through lactoferrin-mediated macrophage targeting 8, as well as in Alzheimer's disease via modulation of the C/EBPβ/AEP pathway and gut microbiota 11. Our preliminary screening of traditional Chinese medicine monomers revealed that PA is a promising anticancer agent, as it strongly inhibits growth in multiple cancer cell lines in a dose- and time-dependent manner. Notably, its pronounced antiproliferative effects in NSCLC cells warrant further mechanistic investigation.

Our experimental data from both *in vitro* and *in vivo* models consistently demonstrated the ability of PA to suppress NSCLC proliferation and xenograft tumor growth. Transmission electron microscopy revealed significant accumulation of autophagosomes and autolysosomes in the PA-treated cells, indicating the induction of autophagy. This observation aligns with established paradigms of autophagy-mediated programmed cell death 18-19, where excessive autophagic flux coincides with apoptotic activation.

The molecular machinery of PA-induced autophagy was further elucidated through biomarker analysis. Beclin-1, as the core component of the VPS34 complex, initiates autophagosome nucleation under stress conditions 27. During LC3 processing, the conversion of LC3-I to membrane-bound LC3-II facilitates autophagosome expansion 28. P62 protein, which mainly participates in the selective degradation process of autophagy, can recognize and bind to ubiquitinated proteins, and transport substrates to autophagosomes for degradation 29. Our findings demonstrate coordinated upregulation of LC3-II and Beclin-1 expression and downregulation of P62 expression, confirming the ability of PA to induce autophagic cell death in NSCLC. This triple-marker validation provides robust evidence for the effect mechanism of PA in NSCLC.

As a natural product, PA has favorable bioavailability and safety profiles. However, its inherent multitarget nature presents technical challenges in identifying its primary therapeutic targets. To systematically elucidate the drug targets of PA responsible for inducing autophagic cell death in NSCLC cells, we employed an integrated experimental approach, including ‌DARTS, molecular docking, and ‌CETSA. Our results demonstrate that GNAI1 is a direct molecular target of PA. GNAI1 functions as the alpha inhibitory subunit of heterotrimeric G proteins and associates with the Gβγ subunit to form a functional G protein complex. Upon ligand activation of cell surface ‌G‌ protein-‌c‌oupled ‌r‌eceptors (GPCRs), GNAI1 binds to the activated receptor. This interaction triggers GDP-to-GTP exchange on GNAI1, leading to its dissociation from the Gβγ subunit 30. Activated GTP-bound GNAI1 (Gαi-GTP) subsequently inhibits ‌a‌denylate ‌c‌yclase (AC) activity, thereby reducing intracellular cyclic adenosine monophosphate (cAMP) levels and suppressing cAMP-dependent protein kinase ‌A‌ (PKA) signaling. cAMP serves as a crucial second messenger, regulating diverse intracellular signaling cascades, including the ERK and STAT3 pathways, thereby influencing cell proliferation, differentiation, and metabolic processes 31-33. Notably, our findings indicate that PA does not significantly alter GNAI1 expression levels. These findings suggest that PA does not modulate the biological function of GNAI1 through transcriptional regulation but likely exerts its effects through direct binding to the GNAI1 protein itself.

The functional realization of GNAI1, as a G protein alpha subunit family member, typically depends on dynamic interactions with GPCRs or scaffold proteins. ARRB1 (β-arrestin1), a widely studied GPCR scaffold protein, co-mediates downstream signaling pathways with GNAI1 through established paradigms 34. Therefore, elucidating the GNAI1-ARRB1 interaction could provide a breakthrough in deciphering the molecular pathways of PA for the regulation of autophagy. ARRB1 functions intracellularly as a downstream GPCR effector to regulate signal transduction processes 35. It interacts with phosphorylated GPCRs to prevent G protein binding and terminate signaling 36 while also initiating new pathways, such as ERK cascade regulation through protein interactions 37-38. Our findings demonstrate that PA induces autophagic cell death in NSCLC by targeting GNAI1 and modulating the GNAI1/ARRB1 complex equilibrium, thereby regulating the ERK and mTOR signaling axes. However, our data also suggested that PA can partly inhibit the JAK2/STAT3 signaling pathway, while the direct target was not GNAI1. Additionally, it is important to note that mTOR is a key protein kinase in autophagy formation, regulated by multiple upstream proteins and signaling pathways 17, 39. Among these, PI3K/AKT is closely associated with autophagy initiation, while P53, AMPK, and MAPK/ERK participate in regulating the autophagy process. Our findings suggest that PA can target GNAI1 to modulate ERK/mTOR, indicating that PA-induced autophagic cell death is closely linked to autophagy regulation but has a relatively minor impact on autophagy initiation. Moreover, unlike the targeting effect of PA on GNAI1 to regulate ERK/mTOR, its modulation of JAK/STAT3 was an unexpected discovery in this study. Therefore, the specific mechanism through which PA inhibits the JAK2/STAT3 signaling pathway requires further in-depth exploration and clarification in the future.

This study preliminarily reveals the great potential of PA as a potential anticancer drug for NSCLC, but the anticancer effect and safety of PA still need further confirmation in real-world clinical application scenarios. In addition, there is still room for improvement in the validation of the animal models in this study. For example, genetically engineered mouse models and patient-derived xenograft models can be utilized to verify the *in vivo* efficacy and safety of PA, further optimize the dosing regimen of PA, and evaluate its antitumor effect and toxic side effects in immunocompetent animal models, providing a basis for clinical trial design. Moreover, novel delivery methods, such as nano-drug delivery, can also be considered, which have led to revolutionary breakthroughs in the clinical application of natural compounds, addressing the inherent pharmacokinetic bottlenecks of natural compounds and significantly enhancing their bioavailability, targeting ability, and safety, thereby transforming them from laboratory potential into practical clinical therapeutic tools.

Our current research focuses primarily on macroautophagy and has not yet delved into specific types of autophagy, such as mitophagy and endoplasmic reticulum (ER) autophagy 40. Mitophagy is a crucial process through which cells selectively eliminate damaged or redundant mitochondria via autophagy mechanisms. It relies primarily on the ubiquitin-dependent PINK1/Parkin pathway and the ubiquitin-independent receptor-mediated pathway 41 and also includes some newly discovered mechanisms, such as TMBIM6-NDUFS4 pathway-mediated mitophagy 42 and nuclear receptor subfamily 4 group A member 1-mediated FUN14 domain containing 1-dependent mitophagy 43. ER autophagy involves mainly the recognition and targeting of ER regions that require degradation by ER autophagy receptors (such as FAM134B, RTN3, and SEC62), connecting them with autophagosomes, and ultimately degrading them in lysosomes 44. Furthermore, an increasing number of scholars have discovered that natural compounds can exert their pharmacological effects by regulating these two types of autophagy. For instance, the ligustrazine nano-drug delivery system mitigates doxorubicin-induced myocardial injury through a mechanism involving the Piezo-type mechanosensitive ion channel component 1-prohibitin 2 pathway, which orchestrates mitochondrial quality surveillance 45. Astragaloside IV alleviates septic myocardial injury through DUSP1-Prohibitin 2-mediated mitochondrial quality control and ER-autophagy 46. These studies provide important research directions for delving into the pharmacological effects of natural compounds.

In addition, this study has several technical limitations: The effect of PA on the GNAI1-ARRB1 complex requires further validation via proximity ligation assay (PLA) 47, and the functional localization of the complex in autophagy requires additional assays (e.g., co-transfection with GFP-LC3/RFP-GNAI1/Cyan-ARRB1 for confocal microscopy-based co-localization analysis with autophagosomes or lysosomal trackers). Future work should include Co-IP and domain mutation experiments to map interaction details, with cryo-EM structural analysis or biased ligand development offering novel intervention strategies.

## Conclusion

**Figure 7:**
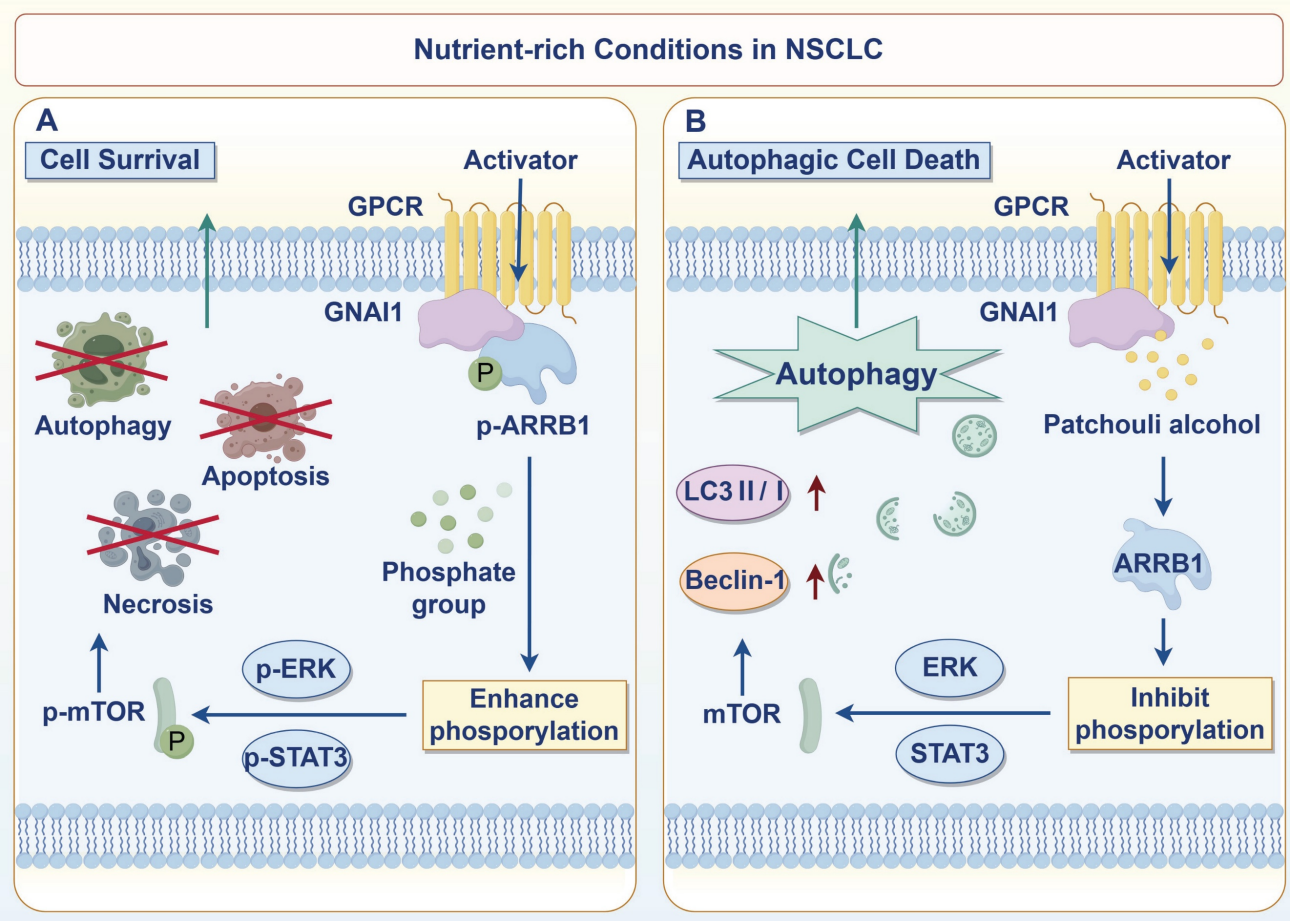
PA induces autophagic cell death in NSCLC cells by targeting GNAI1 to mediate the dissociation of the GNAI1/ARRB1 complex‌.

In conclusion, we revealed that PA targets GNAI1 to dissociate the GNAI1/ARRB1 complex, inhibiting downstream ERK, JAK2/STAT3, and mTOR pathway activation and inducing autophagic cell death in NSCLC. This mechanism enables new therapeutic approaches for NSCLC based on autophagy modulation (Fig. 7).

## Materials and Methods

### CCK-8

Human NSCLC cell lines A549 and H1299 (obtained from ATCC) were cultured in RPMI-1640 supplemented with 10% fetal bovine serum (FBS). Cells were maintained at 37°C in a humidified atmosphere containing 5% CO₂. For cell viability experiments, cells were seeded at a density of 1 × 10³ cells per well in 96-well microplates. Plates were incubated for 24 hours to allow cell attachment before treatment initiation. PA was prepared as a 400 mM stock solution in dimethyl sulfoxide (DMSO). Working concentrations were generated through serial dilution in culture medium, yielding serial concentrations ranging from 1.5625 μM to 400 μM. A vehicle control (0.1% DMSO in medium) was included at each treatment timepoint. Cells were exposed to PA or vehicle control for 24, 48, or 72 hours under standard culture conditions. Cell viability was measured using the Cell Counting Kit-8 (CCK-8) assay. 10 μL of CCK-8 reagent was added to each well, followed by incubation at 37°C for 1-4 hours until color development stabilized. Absorbance was measured at 450 nm using a microplate reader. The inhibition rate was calculated using the formula: Inhibition rate (%) = [(OD treatment group-OD blank group)/(OD control group-OD blank group)] × 100%.

### Colony formation assay

A549 and H1299 cells were seeded at 1,000 cells/well in 6-well plates and treated with specified PA concentrations. Vehicle control (0.1% DMSO) was included. Cells were maintained at 37 °C, 5% CO₂ until colonies formed (typically 7-14 days). Post-incubation, cells were gently washed with PBS, fixed with 4% paraformaldehyde (15 min), washed 3× with PBS, stained with 0.5% crystal violet (30 min), rinsed extensively with deionized water until background clearance. Colonies (defined as ≥ 50 cells) were counted after photographic documentation. Colony formation rate was calculated as: % Colony formation = (Number of colonies / Cells seeded) × 100%.

### Transmission electron microscopy detection

To assess autophagic ultrastructural changes, A549 and H1299 cells were seeded in culture dishes and divided into: Control group (vehicle treatment), PA-treated group (48 hours exposure). Post-treatment, cells were washed 3× gently with PBS, harvested by mechanical scraping in PBS, pelleted via centrifugation (1,000 × g, 5 min), resuspended in 3 mL TEM-grade fixative (e.g., 2.5% glutaraldehyde), fixed at 4°C for 2 hours with gentle agitation. Post-fixation, the cell pellet was washed with PBS and embedded in 1% molten agarose for sectioning support. Use an ultra-thin slicer to cut fixed cell samples into ultra-thin sections of 70-90 nanometers, and place the sections on a platinum plated copper grid. Slices are subjected to comparative staining at room temperature, typically using 1% lead staining solution and 2% urotropin, with a staining time of 10-15 minutes to enhance the contrast of cell structures. After staining, wash the slices with PBS to remove excess staining solution. Finally, the stained sections were observed using transmission electron microscopy (TEM) to observe the morphological changes of autophagosomes, lysosomes, and other related cellular structures. Images were captured at consistent magnifications (10,000-30,000×) for comparative analysis of autophagic features.

### Autophagic flux monitoring via mRFP-GFP-LC3 tandem fluorescence‌

Autophagic flux was assessed using the mRFP-GFP-LC3 tandem reporter system, where GFP fluorescence indicates autophagosomes and mRFP fluorescence persists in acidic autolysosomes. A549 or H1299 cell lines were transfected with GFP-LC3 and RFP-LC3 plasmids, and autophagosomes and autolysosomes were labeled. After transfection, the cells were further cultured for 24 hours to ensure the expression of fluorescent proteins. Subsequently, cells were treated with PA containing media of different concentrations to induce changes in autophagic flux. After processing, wash the cells three times with PBS to remove unbound fluorescent markers, and fix them with 4% paraformaldehyde for 30 minutes. After fixation, wash the cells with PBS and then permeabilize with 0.1% Triton X-100 for 10 minutes to enhance signal permeability. After permeabilization, block the cells with PBS containing 5% BSA for 1 hour to reduce non-specific binding. Next, the cells were incubated at room temperature with antibodies targeting LC3 for 2 hours, and then washed with PBS to remove unbound antibodies. Finally, the fluorescence signals of GFP and RFP were observed under a fluorescence microscope to analyze changes in autophagic flux, and the formation and degradation of autophagosomes were quantitatively evaluated by the ratio of GFP to RFP fluorescence intensity.

### Western blot

Collect cells, add lysis buffer, and lyse on ice for 30 minutes. After quantification using BCA method, add loading buffer and heat for 10 minutes to denature. Perform electrophoresis using 7.5% to 15% SDS-PAGE gel, wet roll the membrane with 220V for 40-90 minutes, seal with 5% BSA at room temperature for 2 hours, thoroughly wash the membrane with TBST, pour out the TBST, add the corresponding primary antibody (1:1000) according to the marker position, and incubate overnight at 4 °C. After washing the membrane with TBST, the corresponding species' secondary antibody (1:10000) was added and incubated at room temperature for 2 hours. After thorough washing with TBST, the membrane was soaked in ECL luminescent solution and imaged using a developing instrument. The grayscale values were statistically analyzed using Image J.

### Subcutaneous xenograft model establishment and PA intervention

SPF grade male BALB/c nude mice (4-6 weeks old, 16-18g) were purchased from Shanghai Jihui Experimental Animal Co., Ltd., with animal qualification certificate number SYXK (Shanghai) 2020-0009. They were housed at the Experimental Animal Center of Shanghai University of Chinese Medicine, with a 12 hours light dark cycle and free access to food and water. This study was approved by the Experimental Animal Ethics Committee of Shanghai University of Traditional Chinese Medicine (Approval No: PZSHUTCM2401180006), and all operations and experiments comply with ethical requirements. The formal experiment will begin after one week of adaptive feeding. After adaptive feeding of male BALB/c nude mice for one week, A549 and H1299 cells were used to prepare a cell suspension of 2 × 10^7^ cells/ml, which was stored on ice. Each nude mouse was inoculated with 0.1 ml of cell suspension under the armpit to establish a subcutaneous transplant tumor model of human lung cancer in nude mice.

After tumor inoculation, the growth of the tumor was monitored daily. When the subcutaneous transplant tumor volume reached 50-100 mm^3^ (average 12 ± 4 days), the tumor bearing mice were randomly divided into 5 groups (n = 5): model group (0.9% NaCl solution containing DMSO), low/medium/high dose group of PA (5, 10, 15 mg/kg/d), and positive control group of rapamycin (2 mg/kg/d). The PA group and rapamycin group were both dissolved in DMSO and diluted with physiological saline, with no more than 1% DMSO in each group. The above groups were injected intraperitoneally with 0.2 mL daily until the end of the experiment.

Tumor volume and body mass measurement: After the start of administration, the length and length of subcutaneous transplanted tumors in nude mice were measured every 4 days using a vernier caliper, and the tumor volume was calculated. Tumor volume = 0.52 × long diameter × short diameter × short diameter, and plot the tumor growth curve. Simultaneously measure the body mass and draw the body mass curve. After a 42 to 49 days dosing cycle, the endpoint of administration has been reached, euthanasia treatment was performed on mice, and tumor tissue was quickly removed, surface adipose tissue was removed, and the tumor mass was measured. Tumor inhibition rate of subcutaneous transplantation in nude mice = (average tumor mass of the model group - average tumor mass of each treatment group)/average tumor mass of the model group × 100%.

### Tissue immunofluorescence detection

Tumor tissue samples were fixed in 4% paraformaldehyde solution and subsequently processed for either paraffin embedding and sectioning (4 μm thickness), or immediate embedding in optimal cutting temperature (OCT) compound for frozen section preparation. Deparaffinization was performed using xylene followed by gradual dehydration through an ethanol gradient series. Antigen retrieval was conducted using sodium citrate buffer (pH 6.0) with heat treatment at 98 °C for 20 minutes. Sections were sealed with blocking solution containing 5% bovine serum albumin (BSA) and 0.3% Triton X-100 at room temperature for 1 hour. Primary antibody incubation was performed overnight at 4 °C. Fluorescently labeled secondary antibodies (Alexa Fluor 488 or 594 conjugates) were applied in the dark at room temperature for 2 hours. After thorough washing, nuclei were counterstained with 4',6-diamidino-2-phenylindole (DAPI). Slides were mounted with anti-fade mounting medium. All the immunofluorescence results are high-resolution panoramic images obtained through the OLYMPUS VS200 digital pathology slide scanner. ImageJ software (National Institutes of Health, USA) was utilized for quantitative analysis of target protein expression.

### RNA sequencing analysis

Sample preparation and RNA extraction: Take logarithmic growth stage NSCLC cells (Experimental group: PA treatment, Control group: DMSO treatment), washed with PBS and lysed with TRIzol reagent, and total RNA was extracted by chloroform layering. Genomic DNA was digested using DNase I (Thermo Fisher, AM2239), RNA purity was detected using NanoDrop 2000 (A260/A280=1.8-2.1), and RNA integrity was evaluated using Agilent 2100 Bioanalyzer (RIN≥8.0). Library construction and quality control: using NEBNext® UltraTM II Directional RNA Library Prep Kit (NEB, E7760) for strand specific library construction: (1) Oligo (dT) magnetic bead enrichment of mRNA, (2) Mg^2+^ ion fragmentation (at 94 °C for 8 min) resulted in a 200-300 bp fragment, (3) Reverse transcription synthesis of cDNA, followed by end repair and connection to sequencing adapters, (4) PCR amplification (15 cycles) index. The quality of the library was quantified by Qubit 4.0 (concentration ≥ 20 ng/μL) and detected by Agilent 2100 (fragment peak±50 bp), and qualified libraries were mixed equimolar. High throughput sequencing: Double ended 150 bp sequencing (PE150) was performed using Illumina.

NovaSeq 6000 platform. Each sample data size is ≥ 6 Gb, quality control standard: Q30 ≥ 85%, effective data ratio≥90%. After FastQC evaluation of the raw data, low-quality bases (SLIDINGWINDOW: 4:20) and adapter sequences were removed using Trimomatic (v0.39). Bioinformatics analysis: (1) Sequence alignment: Hisat2 (v2.2.1) maps Clean reads to the human reference genome (GRCh38), (2) Gene expression quantification: Feature Counts (v2.0.3) were used to calculate FPKM values, (3) Differential gene screening: DESeq2 (v1.34.0) identified genes with |log2FC| ≥ 1 and FDR < 0.05, (4) Functional enrichment: ClusterProfiler (v4.2.2) was used for GO and KEGG pathway analysis.

### TCGA database analysis

Download and organize the RNAseq data for the STAR process of the TCGA-ALL (Pan Cancer) project (https://portal.gdc.cancer.gov), extract TPM format data, and extract corresponding paired adjacent and cancer samples. The data processing method is log2 (value+1), and appropriate statistical methods (stats package and car package) is selected based on the characteristics of the data format for statistical analysis (if the statistical requirements are not met, statistical analysis will not be performed). Visualize the data using the ggplot2 package. Based on survival prognosis data, the optimal cutoff values for GNAI1 and MPZL1 expression were determined using the maximum selection rank statistic, and lung adenocarcinoma (LUAD) patients were divided into high expression and low expression subgroups. Kaplan-Meier survival curves were used to analyze the correlation between the expression levels of GNAI1 or MPZL1 and overall survival (OS). Use Log rank test to compare survival curves between groups, and statistically validate the relationship between GNAI1 or MPZL1 expression and prognosis based on hazard ratio (HR) and 95% confidence interval (CI), in order to evaluate the prognostic value of high and low expression groups of GNAI1 and MPZL1 for OS in lung adenocarcinoma patients. On the other hand, based on the LUAD related data in the TCGA database, the correlation between GNAI1 or ARRB1 and the transcriptome enrichment analysis was analyzed to obtain relevant pathways such as JAK2/STAT3, AMPK/mTOR, PI3K/AKT, MAPK/ERK1/2, p53/mTOR, etc.

### Drug affinity responsive target stability

Using label free Drug Affinity Responsive Target Stability (DARTS) technology to screen potential targets of PA in NSCLC cells. Based on the principle of enhancing protein resistance to protease after drug target binding, A549 cells were divided into a control group (DMSO solvent) and a PA treatment group (effective concentration optimized through pre-experiment). After extracting total protein with non-denaturing lysis buffer (containing protease inhibitor), they were co incubated with gradient concentrations of Pronase (enzyme digestion ratio 1:300, incubation at 37 °C for 30 minutes). By analyzing the protein degradation differences between the two groups through SDS-PAGE, target protein bands with significantly increased stability were screened in the PA treatment group. The peptide sequence was further detected using liquid chromatography tandem mass spectrometry (LC-MS/MS), and the mass spectrometry data was searched using Proteome Discoverer 2.4 software. Candidate targets were identified by combining with the UniProt database.

### Molecular docking

Based on the binding targets selected by DARTS, molecular docking analysis was performed using computer simulation technology to evaluate the binding mode and affinity between PA and the targets. AutoDock Vina 1.1.2 software was used for molecular docking work. Before docking, PyMol 2.5.5 was used to treat the receptor protein, including removing water molecules, salt ions, and small molecules. Then set up a docking box to wrap the entire protein structure. In addition, all processed small molecules and receptor proteins were converted to the PDBQT format required for AutoDock Vina 1.1.2 docking using ADFRSuite 1.0. When docking, the global search detail is set to 32, and the other parameters remain at their default settings. The docking conformation with the highest output score is considered to be the binding conformation, and the docking results were visualized and analyzed using PyMol 2.5.5 and Discovery Studio Visualizer.

### Molecular dynamics simulation

Based on docking results, small molecule protein complexes were obtained as initial structures for all-atomic molecular dynamics (MD) simulations using the AMBER 22 software. Prior to simulation, the partial atomic charges of small molecules were calculated using the antechamber module in conjunction with Hartree-Fock (HF) self-consistent field (SCF) calculations employing the 6-31G* basis set in Gaussian 09 software. Subsequently, small molecules and proteins were parameterized using the GAFF2 force field and ff14SB force field, respectively. Each system underwent hydrogen atom addition using the LEaP module, followed by solvation with a truncated octahedral TIP3P water box extending 10 Å beyond the solute. Neutralization of the system charge was achieved by adding Na⁺ and Cl⁻ ions. Topology and parameter files were then generated for subsequent MD simulations. Following energy minimization, the systems were subjected to gradual heating from 0 K to 298.15 K over 200 ps under constant volume (NVT ensemble) conditions with a controlled heating rate. Subsequently, a 500 ps NVT simulation was performed at 298.15 K to allow for proper solvent equilibration. Equilibration continued with a 500 ps NPT (isothermal-isobaric) simulation at 298.15 K and 1 atm pressure. Finally, production MD simulations were carried out for 100 ns under periodic boundary conditions using the NPT ensemble. During simulations, the non-bonded cutoff distance was set to 10 Å, long-range electrostatic interactions were treated using the Particle Mesh Ewald (PME) method, hydrogen bonds were constrained using the SHAKE algorithm, and temperature control was implemented via the Langevin thermostat with a collision frequency (γ) of 2 ps⁻¹. The integration time step was 2 fs, and trajectory frames were saved every 10 ps for subsequent analysis.

### Cellular thermal stability analysis

Verify the target of PA using Cellular Thermal Stability Analysis (CETSA) method. After treating A549 and H1299 cells with different concentrations of PA, the cells were collected and lysed to obtain cell lysate. Subsequently, the cell lysate was divided into multiple small tubes and incubated at a series of temperatures (such as 37 °C, 42 °C, 47 °C, etc.) for a certain period of time to extract proteins. The protein samples were separated by SDS-PAGE electrophoresis and transferred onto PVDF membranes. Subsequently, the target protein was incubated with specific antibodies and subjected to chemiluminescence detection after binding with secondary antibodies. By comparing the expression changes of target proteins at different temperatures, the interaction between PA and target proteins can be further confirmed.

### Generation of GNAI1 and ARRB1 stable knockdown cell lines‌

Stable knockdown NSCLC cell lines for GNAI1 and ARRB1 were generated using lentiviral shRNA delivery with the following protocol. The specific steps are as follows: design shRNA sequences targeting the target genes GNAI1 and ARRB1 and insert them into lentiviral vectors. Co-transfect packaging plasmids, envelope plasmids, and lentiviral vectors with 293T cells. Collect the supernatant 48-72 hours later, centrifuge and filter to obtain virus particles, and measure the virus titer by qPCR. Based on virus titer calculation, pre-experiment was performed to screen multiplicity of infection (MOI). Inoculate A549 cells into a 24 well plate (density 40%~50%), add lentivirus solution and 5 μg/mL Polybrene at MOI = 10 to enhance infection, incubate for 12-24 hours, replace the culture medium, and observe GFP expression under a fluorescence microscope for 48 hours to evaluate infection efficiency. The H1299 cell operation is the same as above, MOI = 20. After infection, cells were passaged and screened using gradient puromycin (gradually increasing concentration) to obtain stable knockdown strains. The expression level of the target protein was verified by Western Blot, and monoclonal cell lines were screened using limited dilution method.

### RNA extraction and quantitative PCR analysis‌

Total RNA was extracted from samples using TRIzol reagent (Invitrogen) following the manufacturer's protocol. RNA concentrations were quantified using a NanoDrop ND-1000 spectrophotometer, and aliquots of each sample were immediately used for cDNA synthesis. For each sample, 500 ng of total RNA was reverse transcribed into cDNA using the PrimeScript RT Reagent Kit (TaKaRa). Relative mRNA expression levels of target genes were quantified by qPCR using SYBR Premix Ex Taq (TaKaRa). GAPDH mRNA was employed as an internal control for normalization. Reactions were performed on an ABI 7500 Real-Time PCR System (Applied Biosystems). The threshold cycle (Ct) values were determined, and relative expression levels were calculated using the comparative Ct method (2^-△△Ct^ method).

### Prediction of GNAI1 interacting proteins

To predict proteins that interact with GNAI1, we performed bioinformatics analysis using the STRING database (https://string-db.org).

### Protein-protein docking assay of GNAI-ARRB1

The three-dimensional structural files of GNAI1 and ARRB1 proteins were separately retrieved from the AlphaFold database. Structural preprocessing was performed using PyMOL 2.5.3, during which regions with low prediction confidence or structural errors were removed. Global rigid-body docking was conducted using ZDOCK 3.0.2 to predict the binding mode between GNAI1 and ARRB1. The docking procedure was executed with the software's default configuration to comprehensively sample potential binding conformations. Following docking, the obtained protein complex was subjected to energy minimization using AMBER 18. Minimization was performed under the ff14SB force field to obtain a more physically realistic three-dimensional conformation. The energy-minimized complex conformation was submitted to the ‌PRODIGY web server‌ (https://wenmr.science.uu.nl/prodigy/) for binding affinity prediction. According to the platform description, PRODIGY (PROtein binDIng enerGY prediction) is a web service focused on predicting binding affinity in biological complexes, particularly for ‌protein-protein interactions‌. It accepts input in PDB or mmCIF format and returns estimated binding free energy (ΔG) based on structural features. The conformation with the most favorable predicted binding energy was selected as the optimal binding mode. Finally, the selected optimal binding mode was visualized and analyzed using PyMOL 2.5.3 to examine interface residues, hydrogen bonding, hydrophobic contacts, and other key interaction features.

### Co-immunoprecipitation

To verify the interaction between endogenous GNAI1 and ARRB1, a magnetic bead co-immunoprecipitation (Co-IP) experiment was conducted. The steps are as follows: Collect non-small cell lung cancer cells, wash them with pre-cooled PBS, add RIPA lysis buffer containing protease inhibitor and lyse them on ice for 30 minutes, centrifuge to obtain the supernatant and quantify the protein concentration. Take an equal amount of total protein and add anti-GNAI1 or anti-ARRB1 antibodies (experimental group) or isotype IgG (negative control group) to different histones. Incubate overnight at 4 °C, then add pre-equilibrated Protein A/G magnetic beads and continue incubation for 2 hours. After gently washing the magnetic beads with detergent for 4 times, add SDS loading buffer and boil to elute the protein complex. Western blot was used to detect the expression levels of ARRB1 and GNAI1 in the elution products, while Input was used as a control to verify specific interactions.

### Cellular immunofluorescence

A549 and H1299 cells in logarithmic growth phase were inoculated into a 24 well confocal plate at a density of 1 × 10^4^/0.5mL. After the cells have stabilized and adhered to the wall, they are fixed, permeabilized, sealed, and subjected to the first and second rounds of immunofluorescence staining. Dilute the target proteins GNAI1 and ARRB1 at a ratio of 1:500. Add GNAI1 antibody in the first round of incubation and incubate overnight at 4 °C. Add Alexa Fluor 488 labeled fluorescent secondary antibody the next day. For the detection of ARRB1, repeat the above steps, incubate with the second round of primary antibody at 4 °C overnight, and add Cy3 labeled fluorescent secondary antibody the next day. After the staining is completed, DAPI nuclear staining and encapsulation are performed. Finally, under a confocal microscope, a suitable excitation light source is selected based on the excitation wavelength of the fluorescent dye DAPI (ex: 405 nm, em: 460 nm), Alexa Fluor 488 (ex: 488 nm, em: 520 nm), Cy3 (ex: 561 nm, em: 570 nm) and perform image acquisition.

### Statistical analysis

Statistical analysis was conducted using IBM SPSS Statistics 24.0 software. All data were evaluated for normality using Kolmogorov Smirnov test. If the data conforms to a normal distribution, mean ± standard deviation (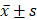) is used for description, and independent sample t-test (two tailed) is used for two group comparison or one-way analysis of variance (ANOVA, two tailed) is used for multi group comparison between groups. If the data does not follow a normal distribution, attempt to perform a natural logarithmic transformation and retest for normality. If the transformed data follows a normal distribution, continue to use a two tailed t-test or ANOVA for analysis. If it still does not follow a normal distribution, describe it using the median (interquartile range), and compare between groups using the Mann Whitney U test (two tailed) for two group comparisons or the Kruskal Wallis test (two tailed) for multi group comparisons. Pearson correlation test (two tailed) was used to evaluate the correlation between variables in the correlation analysis. All statistical tests were two-tailed with significance threshold set at *P* < 0.05.

## Supplementary Material

Supplementary figures and tables.

## Data Availability

All the data generated during this research were included within the article.
