# Supplementary material for: Patchouli alcohol triggers autophagic cell death in non-small cell lung cancer cells through targeting GNAI1 to dissociate the GNAI1/ARRB1 complex
Source: Int J Biol Sci. 2026 Mar 30;22(8):4004–24. doi: 10.7150/ijbs.125690 (PMC13137856; doi:10.7150/ijbs.125690)
Supplement: Supplementary file 1 — Supplementary figures and tables. [file ijbsv22p4004s1.pdf]

Supporting Information for

Original article

Patchouli alcohol triggers autophagic cell death in non-small cell lung cancer cells through targeting GNAI1 to dissociate the GNAI1/ARRB1 complex

Sheng Zhang[2,†], Lei Tang[3,†], Yunzhou Pu[3,†], Yu You[4,†], Hongyu Chen[6], Yongqi Chen[3], Yanqing Huang[6], Xiaodie Liu[3], Qing Song[5]*, Qing Ji[3]*, Liu Yang[1,4,6,*]

[1]Shanghai Baoshan District Wusong Central Hospital (Zhongshan Hospital Wusong Branch, Fudan University), Shanghai 200940, China

[2]Guangdong Lung Cancer Institute, Guangdong Provincial People's Hospital (Guangdong Academy of Medical Sciences), Southern Medical University, Guangzhou 510080, China; Guangdong Provincial Key Laboratory of Translational Medicine in Lung Cancer, Guangdong Provincial People's Hospital (Guangdong Academy of Medical Sciences), Southern Medical University, Guangzhou 510080, China

[3]Department of Medical Oncology & Cancer Institute of Integrative Medicine, Shuguang Hospital, Shanghai University of Traditional Chinese Medicine, Shanghai 201203, China

[4]Chengdu University of Traditional Chinese Medicine, Chendu 610075, China

[5]Department of Medical Oncology, Suzhou TCM Hospital Affiliated to Nanjing University of Chinese Medicine, Suzhou 215007, China

[6]Department of Oncology, Baoshan Hospital Affiliated to Shanghai University of Traditional Chinese Medicine, Shanghai 201999, China

[†]These authors made equal contributions to this work.

*Corresponding authors:

Liu Yang, bsyykyc@shutcm.edu.cn; Qing Ji, qingji1004@shutcm.edu.cn;

Qing Song, songqing12016084@163.com.

Supplementary Figures

Supplementary Figure 1

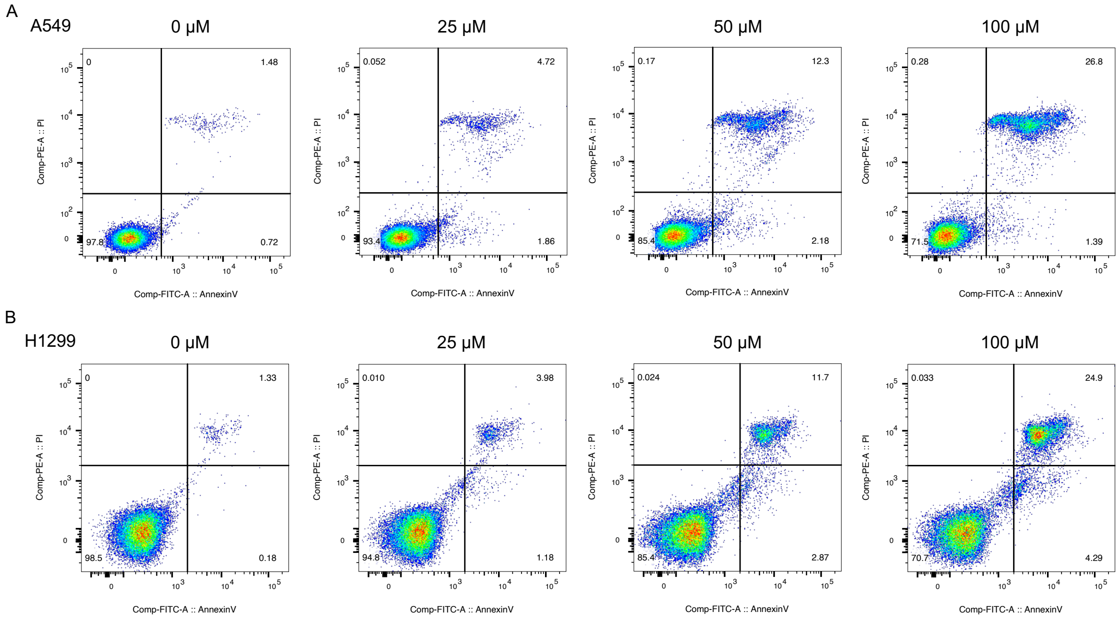

Figure S1 PA partially induces apoptosis in NSCLC cells. (A-B) Flow cytometry was applied to detect the effect of PA on the apoptosis of NSCLC cell lines A549 and H1299.

Supplementary Figure 2

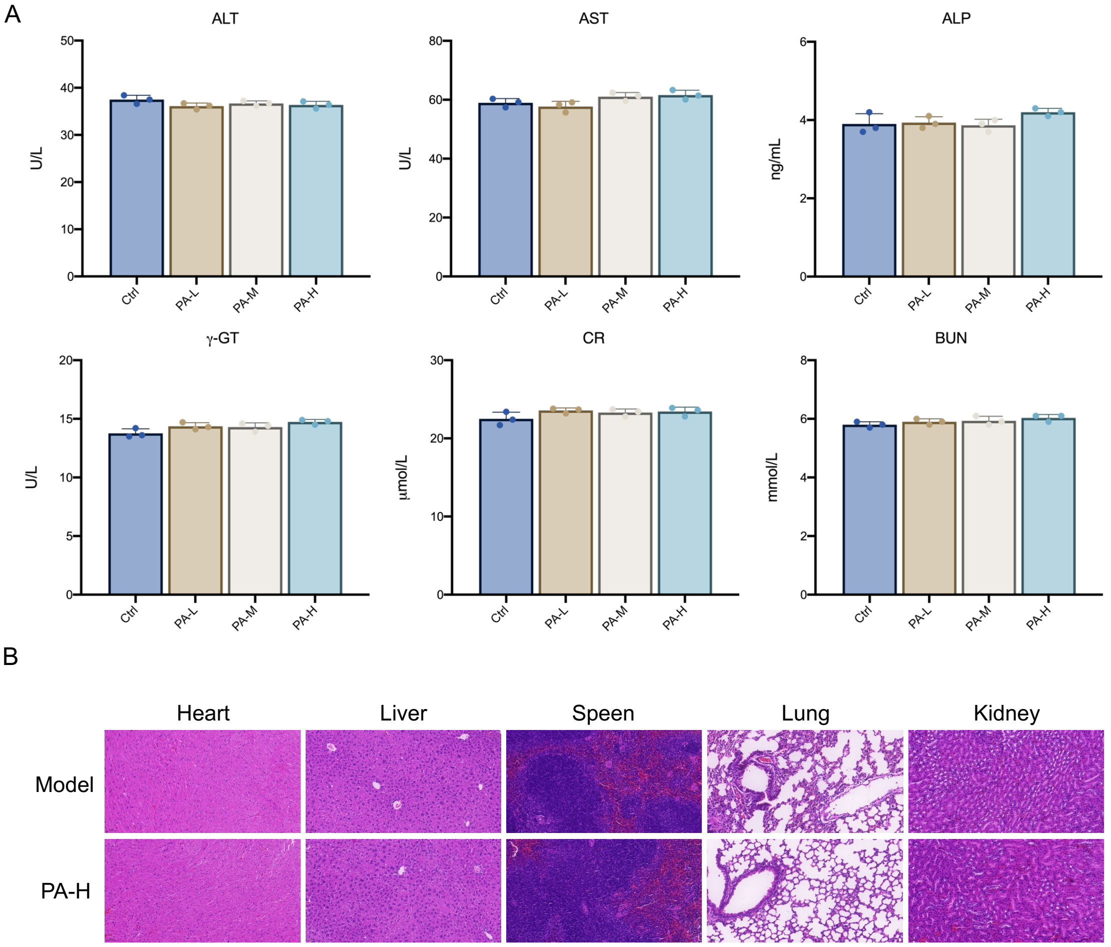

Figure S2 Evaluation of side effects of PA on mice hepatic function. (A) Biochemical analyses of hepatic parameters were performed. (B) HE staining was used to observe the effects of PA on the heart, liver, spleen, lungs, and kidneys of mice.

Supplementary Figure 3

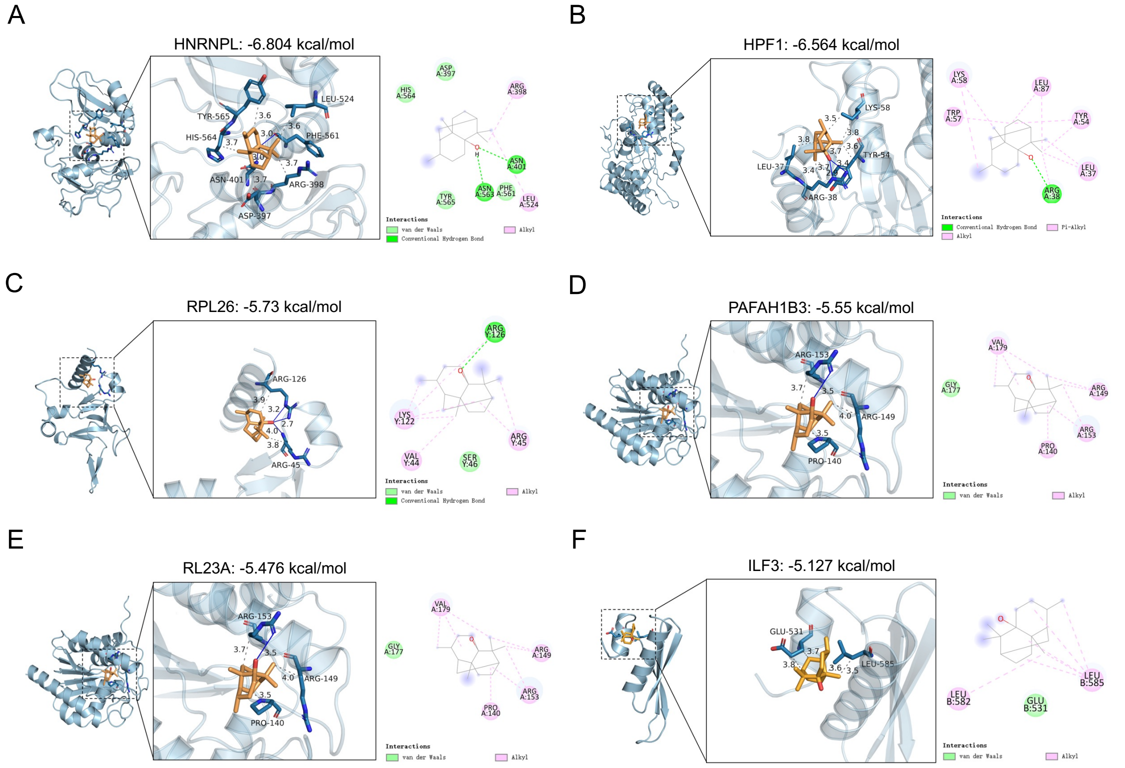

Figure S3 Molecular docking results of PA with six other potential drug targets. A: HNRNPL, B: HPF1, C: RPL26, D: PAFAH1B3, E: RL23A F: ILF3.

Supplementary Figure 4

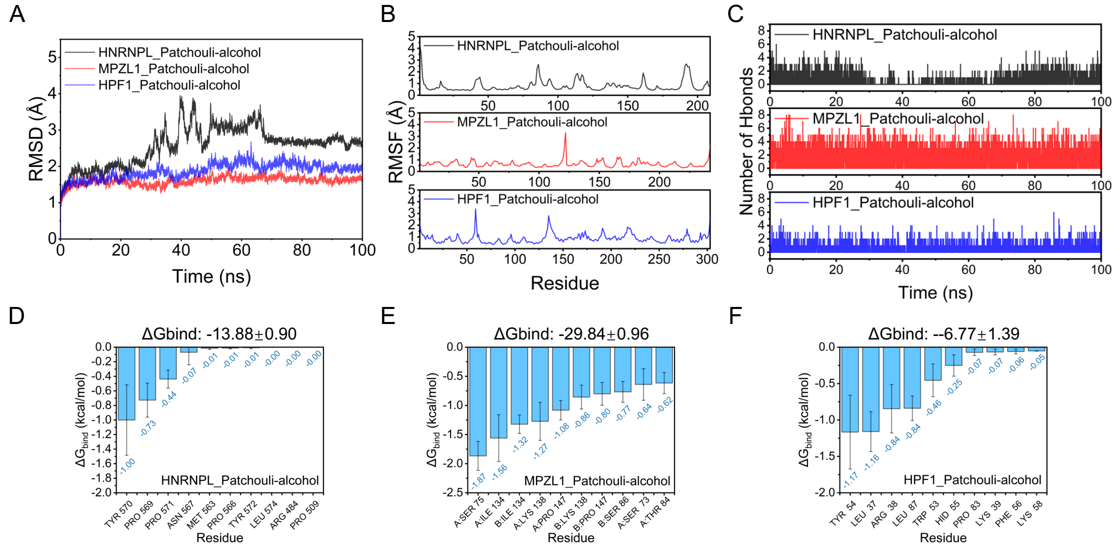

Figure S4 Molecular dynamics simulation and binding free energy analysis results of PA with HNRNPL, MPZL1 and HPF1. (A-C) Molecular dynamics simulation results of PA with HNRNPL, MPZL1 and HPF1. (D-F) The binding free energy analysis results of PA with HNRNPL, MPZL1 and HPF1.

Supplementary Figure 5

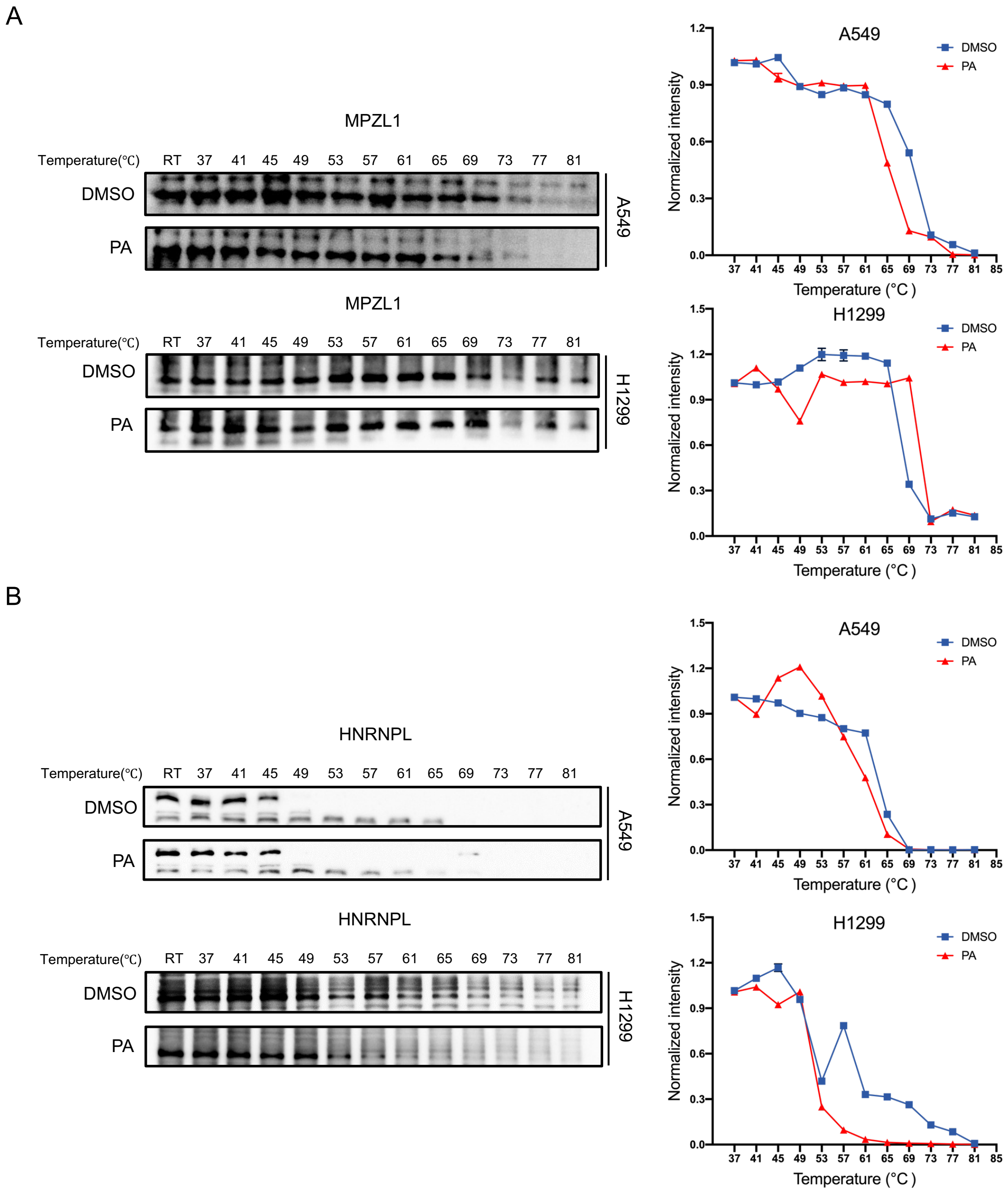

Figure S5 CETSA experiment verifies the binding effect between PA and MPZL1 or HNRNPL. (A) CETSA experiment verifies the binding effect between PA and MPZL1. (B) CETSA experiment verifies the binding effect between PA and HNRNPL.

Supplementary Figure 6

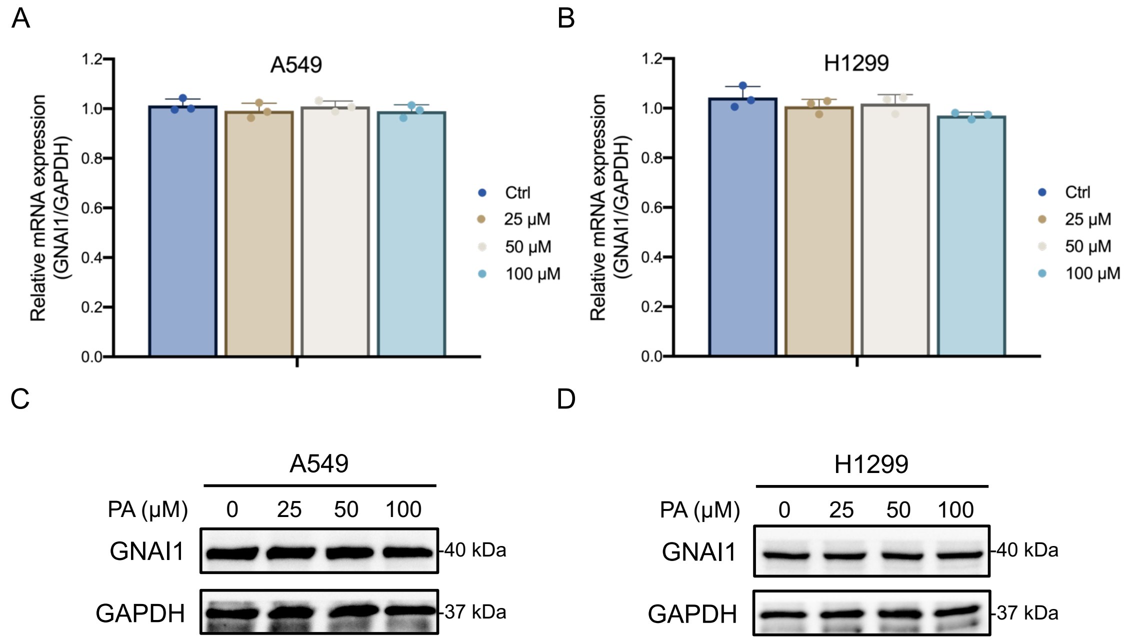

Figure S6 qPCR and WB detection of the effect of PA on GNAI1 expression in A549 and H1299 cells. (A-B) qPCR detection of the effect of PA on GNAI1 mRNA expression in A549 and H1299 cells. (C-D) WB detection of the effect of PA on the expression of GNAI1 protein in A549 and H1299 cells.

Supplementary Figure 7

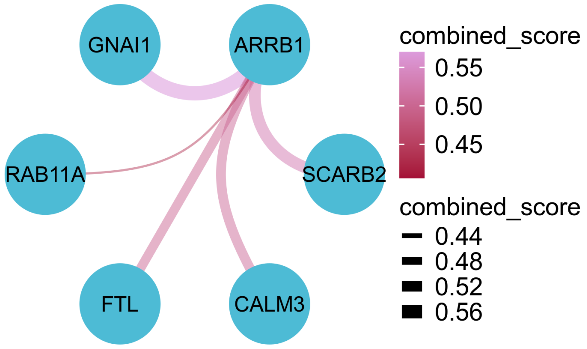

Figure S7 Predicting protein interactions between GNAI1 and ARRB1 based on the String website.

Supplementary Figure 8

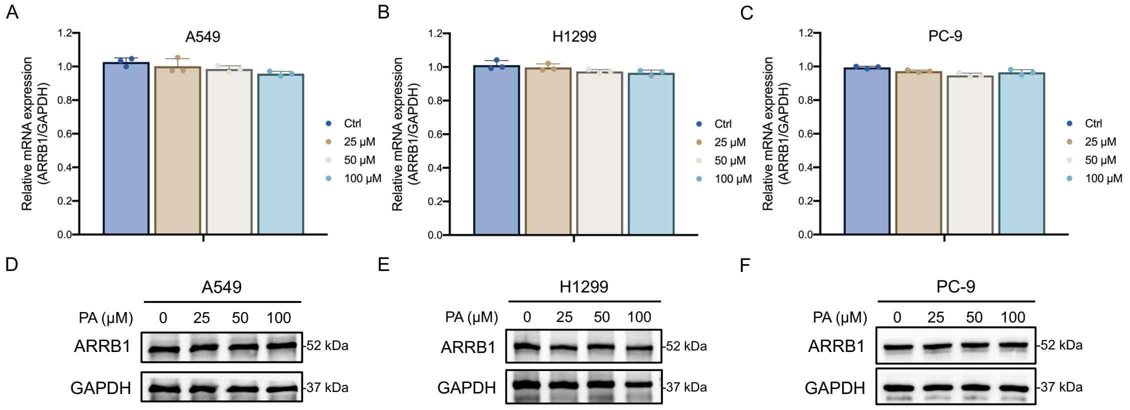

Figure S8 qPCR and WB detection of the effect of PA on ARRB1 expression in A549, H1299, and PC-9 cells. (A-C) qPCR detection of the effect of PA on ARRB1 mRNA expression in A549, H1299, and PC-9 cells. (D-F) Western blot detection of the effect of PA on ARRB1 protein expression in A549, H1299, and PC-9 cells.

Supplementary Figure 9

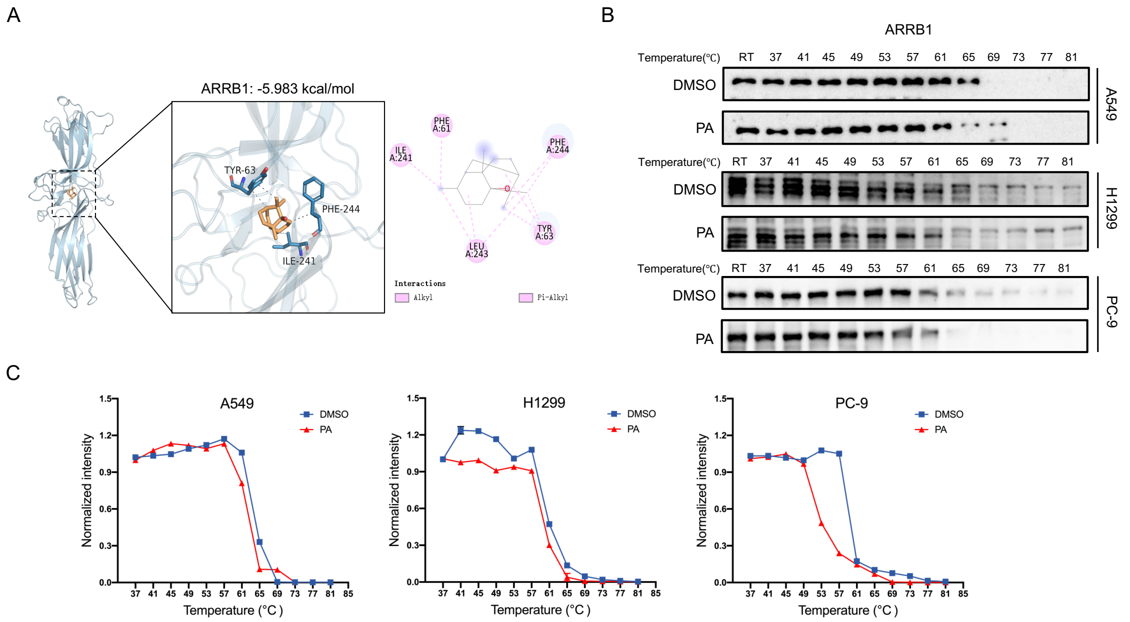

Figure S9 Molecular docking and CETSA experiment results of PA and ARRB1. (A) Molecular docking results of PA and ARRB1. (B-C) CETSA experiment verifies the binding of PA to ARRB1 in A549, H1299, and PC-9 cells.

Supplementary Tables

Supplementary Table 1 Antibodies for WB analysis

Antibodies	Source	Catalog no.
LC3A/B	CST	12741
Beclin 1	CST	3495
GNAI1	CST	5290
MPZL1	Proteintech	29784-1-AP
HNRNPL	Proteintech	18354-1-AP
ARRB1	CST	12697
p-JAK2	CST	3776
JAK2	CST	3230
p-STAT3	Proteintech	28945-1-AP
STAT3	Proteintech	51076-2-AP
p-ERK	Proteintech	28733-1-AP
ERK	Proteintech	11257-1-AP
p-mTOR	Proteintech	28879-1-AP
mTOR	Proteintech	28273-1-AP
p-PI3K	CST	17366
PI3K	CST	4292
p-AKT	CST	4060
AKT	CST	9272
GAPDH	CST	2118

Supplementary Table 2 Antibodies for IF analysis

Antibodies	Source	Catalog no.
LC3A/B	CST	12741
Beclin 1	Santa Cruz	sc-48381
GNAI1	Santa Cruz	sc-13533
ARRB1	Santa Cruz	sc-53780

Supplementary Table 3. The primer sequences for qPCR.

Gene	Primer sequences
GNAI1	F: 5-ATGCACGCCAACTCTTTGTG-3
	R: 5-AGCTGGTACTCTCGGGATCT-3
ARRB1	F: 5-CAAAGGGACCCGAGTGTTCA-3
	R: 5-GCAGGTCAGCGTCACATAGA-3
GAPDH	F: 5-GGTGGTCTCCTCTGACTTCAACA-3
	R: 5-CCAAATTCGTTGTCATACCAGGAAATG-3

Supplementary Table 4. The shRNA sequences for GNAI1.

Gene	Target sequence
shRNA-NC	CCUAAGGUUAAGUCGCCCUCG
shRNA-1	CAGUUUGAAGACCUCAAUAAA
shRNA-2	CAAACCAAAUGAGUACUUAUA
shRNA-3	UUAAAGCUGGGCUCUAGUAUA

Supplementary Table 5. The shRNA sequences for ARRB1.

Gene	Target sequence
shRNA-NC	UUCUCCGAACGUGUCACGU
shRNA-1	GAACUGCCCUUCACCCUAAUGUU
shRNA-2	CGACGUUCUGCAAGGUCUAUU
shRNA-3	UCUGGAUAAGGAGAUCUAUUA
